# Applications of Reinforcement Learning for Autonomous Surgical Robotics: A Systematic Review

**DOI:** 10.3390/biomimetics11080577

**Published:** 2026-08-12

**Authors:** Muhammad Shahid, Abdullah, Zulaikha Fatima, Wasif Feroze, Miguel Jesús Torres Ruiz, Magdalena Saldaña-Pérez, Carlos Guzmán Sánchez-Mejorada, Rolando Quintero Tellez

**Affiliations:** 1 Center for Computing Research, Instituto Politécnico Nacional, Mexico City 07738, Mexico; mshahid.bulc@bahria.edu.pk (M.S.); abdullah2025@cic.ipn.mx (A.); cmejorada@cic.ipn.mx (C.G.S.-M.); quintero@cic.ipn.mx (R.Q.T.); 2 Department of Computer Sciences, Bahria University, Lahore 54600, Pakistan; 3 Faculty of Allied Health Sciences, Superior University, Lahore 54000, Pakistan; su91-bmitm-f23-248@superior.edu.pk; 4 Department of Computer Science, National University of Sciences and Technology, Balochistan Campus, Quetta 87300, Pakistan; wasif.feroze@nbc.nust.edu.pk

**Keywords:** reinforcement learning, surgical robotics, autonomous surgery, sim-to-real transfer, PRISMA 2020, safety-constrained reinforcement learning, vision–language–action models, hierarchical reinforcement learning

## Abstract

Reinforcement learning (RL) has emerged as a promising approach for autonomous surgical robotic subtasks. Recent advances include deep reinforcement learning (DRL), imitation learning (IL), and vision–language–action (VLA) models. However, current evidence remains fragmented across simulation benchmarks, task-specific demonstrations, and limited clinical studies. Existing reviews primarily focus on RL algorithms, while the broader pathway from algorithm development to clinically deployable surgical autonomy has not been comprehensively synthesised. This PRISMA 2020-guided systematic review examines RL, IL, safe RL, simulation-to-real (sim-to-real) transfer, foundation models, VLA systems, and regulatory readiness in surgical robotics. We searched IEEE Xplore, PubMed/MEDLINE, Embase, Scopus, Web of Science, the Cochrane Library, ACM Digital Library, arXiv, and medRxiv for studies published between January 2015 and March 2026, with additional studies identified through backward citation tracing. Eligible studies proposed novel RL, imitation learning, or foundation-model approaches for surgical robotics with empirical validation in simulation or on physical robotic platforms. Two reviewers independently extracted data using a predefined coding scheme, and a third reviewer resolved disagreements. Owing to substantial heterogeneity in platforms, tasks, and outcome measures, a quantitative meta-analysis was not feasible; therefore, the evidence was synthesised narratively using a comparative framework. A total of 220 studies met the inclusion criteria, covering eleven active surgical RL platforms, seven paired sim-to-real studies, emerging foundation-model architectures, and three FDA-cleared robotic systems exhibiting Level 3 autonomy. Available comparative studies suggest that hierarchical approaches can outperform flat policies in long-horizon tasks, while language-conditioned models demonstrated promising multi-step surgical capabilities. Seven paired simulation-to-real studies were identified, encompassing tissue retraction, guidewire navigation, and surgical cutting tasks. Sim-to-real performance gaps varied substantially by task and metric, with success-rate gaps ranging from −10 to 50 percentage points (negative values indicating better real-world than simulated performance), while paired mean spatial errors differed by at most 0.61 mm. Most studies employed domain randomization or visual domain adaptation; hierarchical reinforcement learning demonstrated advantages over flat policies in multi-step surgical tasks. Explicit safety-constrained methods (CPO, CBF, and SER), formal verification, and regulatory-aligned evaluation were reported in fewer than 3% of applied studies. Most evidence remained simulation-based, with no reported autonomous RL execution in vivo in humans. Overall, RL-based surgical robotics appears mature at the simulation stage but remains preclinical for autonomous clinical deployment. Future progress requires stronger sim-to-real validation, multimodal safety-aware architectures, alignment with IEC 62304, ISO 14971, FDA guidance, and the EU AI Act, and open benchmarks that jointly evaluate performance, safety, and surgeon trust.

## 1. Introduction

Surgical robotics emerged from the convergence of minimally invasive surgery (MIS) and robotic engineering, with early platforms such as the da Vinci Surgical System establishing the defining paradigm of master slave telemanipulation: enhanced dexterity, three-dimensional visualisation, and tremor filtration, but without any degree of machine autonomy [1,2,3,4]. These systems transformed operative technique, enabling surgeons to perform complex pelvic, urological, and thoracic procedures through millimetre-scale incisions [5,6]; yet, they remained fundamentally passive instruments, amplifying human skill rather than substituting it [7,8]. A critical intermediate stage followed: the integration of AI-driven analytics into the robotic workflow. Video, kinematic, and force-sensor data streams from robotic platforms enabled machine learning systems to perform objective skill assessment, surgical phase recognition, and workflow analysis, establishing a layer of ‘cognitive assistance’ that went beyond mere instrumentation [3,7,9]. Contemporary AI-aided surgical robotic systems (SRSs) have advanced further still, integrating perception, planning, and control modules capable of partially automating discrete subtasks: soft-tissue cutting and tensioning, suture placement, tissue retraction, haemostatic suction, and deformable object manipulation [2,8,10,11,12,13,14]. This progression from passive tool to analytics platform to partially autonomous agent defines the evolutionary arc that this review systematically analyses.

Within the framework of bioinspired robotics and intelligent motion systems, the surgical environment represents a uniquely challenging domain characterised by the dynamic interplay between rigid instruments and compliant, deformable biological structures [2,11]. Traditional deterministic control paradigms frequently struggle to capture the non-linear mechanics and unpredictable variability inherent in human tissue manipulation [15,16]. In contrast, the application of reinforcement learning within surgical robotic systems (SRSs) establishes a pathway for adaptive robotic behaviour that reflects the principles of biological intelligence and the mechanisms of human motor skill acquisition [7,9]. By framing surgical subtasks as problems of sensorimotor adaptation and perception–action coupling, this review identifies a paradigm shift toward embodied surgical intelligence [13,17]. These autonomous agents are designed to function as physiologically aligned modulators capable of navigating the complex constraints of human–robot collaboration [18,19]. Consequently, analysing the RL-driven pipeline provides critical insights into the development of adaptive robotic systems that mimic the dexterity and robust performance of motile organisms when interacting with dynamic, nature-derived environments.

From a biomimetics perspective, reinforcement learning offers more than a control-engineering convenience: it recapitulates the trial-and-error, reward-driven mechanism by which biological organisms acquire skilled motor behaviour. Surgeons, like any biological agent refining a motor skill, do not learn suturing or tissue handling from an explicit dynamical model of tissue mechanics; they learn through repeated practice, proprioceptive feedback, and outcome-contingent reinforcement, gradually shaping internal representations that generalise across anatomical variability. RL-driven surgical agents are designed to emulate precisely this biological learning loop, substituting a reward signal for the surgeon’s own sense of successful task completion. In this sense, the RL formulations reviewed here are not merely borrowed from general robotics but are a direct computational analogue of the sensorimotor adaptation strategies observed in animals and humans, positioning surgical RL within the broader biomimetic tradition of engineering systems that learn as biological systems do, rather than systems that are explicitly programmed.

This biomimetic parallel extends beyond the learning rule to the architecture of surgical autonomy itself. Hierarchical reinforcement learning, in which a manager policy sequences reusable low-level sub-skills, mirrors the layered organisation of biological motor control, where high-level goal planning in the central nervous system is decoupled from low-level reflexive and rhythmic motor primitives executed by spinal and peripheral circuits. Similarly, safe-RL mechanisms that constrain exploratory actions within a learned safe manifold parallel the protective reflexes and proprioceptive safeguards that prevent biological organisms from generating self-injurious motion during skill acquisition. Vision–language–action models, which ground high-level semantic intent in low-level continuous actuation, likewise echo the integration of cognitive and sensorimotor pathways in biological perception–action loops. Framing surgical RL through this biomimetic lens clarifies why hierarchical, safety-constrained, and multimodal architectures consistently outperform flat, unconstrained policies in the studies reviewed here: they are, in effect, better approximations of the layered, safeguarded, and multimodal control strategies that biological motor systems have evolved to solve comparable problems of dexterous, safety–critical manipulation.

Surgical autonomy is characterised by a hierarchical scale ranging from Level 0 (no autonomy) to Level 5 (full autonomy), a framework originally proposed by Yang et al. [20] and subsequently extended through ISO/IEC standardisation efforts and empirical refinement [15,20,21]. Current clinical deployment is overwhelmingly situated at Level 1 (Robot Assistance); a systematic review of 49 FDA-cleared surgical robots (2015–2023) confirmed that 86% remain at this tier, with only 6% reaching Level 3 (Conditional Autonomy), and no clinically deployed Level 4 or Level 5 device yet identified. The only FDA-cleared systems classified as Level 3 (Conditional Autonomy) were the iSR’obot Mona Lisa 2.0, TSolution One, and ARTAS iX System. [22]. The AquaBeam Robotic System for aquablation of the prostate, the first FDA-cleared robot providing autonomous tissue removal, represents Level 2 (Task Autonomy), autonomously executing a surgeon-defined treatment plan within a tightly constrained procedural domain [22,23]. Research systems predominantly target Levels 2 and 3 in soft-tissue environments, where the deterministic assumptions of orthopaedic robotics are insufficient [15,24,25]. Critically, a growing body of expert opinion argues that the framing of autonomy as a progression toward ‘replacing’ the surgeon is both clinically inappropriate and strategically mistaken. Battaglia et al. [18] contend that understanding surgeon intent, behaviour, and cognitive limitations is the foundational design requirement for the next generation of surgical robot systems that enhance rather than supplant human expertise. Knudsen et al. [19] extend this to a clinical applications framework in which AI–robot systems are evaluated on their capacity for collaborative, adaptive autonomy with surgeons in the loop. This review adopts that philosophy: autonomy is a design property to be calibrated, not a destination to be reached.

Conventional control methods, including rule-based logic and classical model predictive control (MPC), struggle with the high-dimensional state spaces and non-linear deformations characteristic of soft-tissue manipulation [16,26]. Reinforcement learning (RL) addresses this by learning control policies directly from trial-and-error interaction with simulated environments, avoiding the need to explicitly model tissue mechanics or anatomical variability [2,27]. Unlike supervised learning, which requires large annotated expert datasets that are structurally scarce in surgical contexts, RL can iteratively refine behaviour through reward signals aligned with surgical objectives [28,29]. Empirically, RL has been applied across a widening range of surgical subtasks: autonomous tissue cutting and tensioning via evolutionary-augmented deep RL [10]; sim-to-real transfer for internal soft-tissue point positioning on physical dVRK hardware [14]; deformable object retraction and manipulation with domain-adapted visual policies [11]; integration into virtual robotic surgical simulation for suturing motion generation [30]; and cooperative multi-robot assistance through multi-agent RL frameworks [31]. Safe RL formulations explicitly constraining the policy to avoid tissue damage and instrument collision during exploration have demonstrated the feasibility of learning task-automation policies that satisfy hard safety bounds without prohibitive computational overhead [12]. RL thus addresses three properties that alternative paradigms cannot simultaneously provide: adaptability to deformable tissue variability, scalability beyond hand-crafted controllers, and capacity for sim-to-real transfer in safety–critical settings [2,8,12,14]. The persistent vulnerability is reward misspecification, colloquially termed ‘reward hacking ’, wherein agents exploit unintended shortcuts that satisfy the mathematical reward while violating clinical intent [32].

The majority of published RL systems learn task-specific policies: a single skill (suturing segment, peg transfer, needle driving) trained for a fixed setup and platform [10,14,30,31]. These systems exhibit poor generalisation across procedures, instrument configurations, and anatomical variability [8,27]. The emerging response is embodied surgical intelligence: multi-task, multi-modal vision–language–action (VLA) models trained on diverse simulators and datasets, capable of zero-shot transfer across contexts. Long et al. [13] demonstrated surgical embodied intelligence for generalised task autonomy in laparoscopic robot-assisted surgery, achieving multi-task skill training and assistive task execution across different laparoscopic platforms, a landmark result published in Science Robotics. Schmidgall et al. [17] formalised the theoretical and architectural basis for general-purpose foundation models for increased surgical autonomy, published in Nature Machine Intelligence [17]. By integrating vision–language models (VLMs) with hierarchical reinforcement learning (HRL), researchers are composing complex surgical procedures from libraries of reusable skill primitives [17,33,34]. Models such as BiomedGPT leverage large-scale pretraining on diverse biomedical datasets to build unified visual–textual representations enabling cross-task generalisation [35]. Nonetheless, direct empirical evidence for cross-specialty generalisation remains limited and preliminary [34,36], and the sim-to-real gap—the failure of policies trained on curated simulation data to capture microscopic anatomical variability in live procedures—remains the primary translational barrier [37,38].

### Scope and Contributions of This Review

This review provides a systematic analysis of the RL-driven autonomy pipeline in surgical robotics, spanning perception, control, safety, and clinical validation infrastructure, within a PRISMA-guided systematic framework [27,39]. Unlike prior surveys that address deep learning in surgery broadly [39,40], this work specifically targets the interplay between RL algorithms, sim-to-real transfer methodology, and the emerging foundation model landscape. The contributions are threefold: (1) a qualitative synthesis of RL algorithm adoption trends and performance across soft-tissue and hard-tissue surgical tasks, supported by paper-specific evidence presented in structured tables; (2) a critical comparative evaluation of 2021–2026 simulation platforms and their translational fidelity; and (3) a structured roadmap for addressing the Safety Gap that currently prevents autonomous surgical systems from achieving regulatory clearance. The review is organised around four Research Questions: (RQ1) What surgical subtasks have been automated using RL, and with what performance and safety profiles [41]? (RQ2) How effective are current sim-to-real and safe RL strategies under realistic surgical conditions? (RQ3) What is the role of foundation models and VLMs as reward priors and perception backbones for surgical RL? (RQ4) What architectural and data requirements are emerging for generalist, multi-task surgical agents?

## 2. Systematic Review Methodology (PRISMA 2020)

The methodology for this review adheres to the Preferred Reporting Items for Systematic Reviews and Meta-Analyses (PRISMA 2020) guidelines [42,43,44,45]. Given the rapid convergence of foundation models and reinforcement learning (RL) in surgical robotics, this section defines a rigorous, reproducible framework for identifying, screening, and synthesising high-impact literature at the intersection of robotics, artificial intelligence, and clinical surgery.

A structured and predefined review plan was used to guide study identification, screening, eligibility assessment, and synthesis, ensuring methodological consistency and transparency throughout the review process [42,43,44,45].

### 2.1. Research Questions (RQs)

The review is structured around four Research Questions (RQs) targeting distinct technical and translational challenges of AI/RL in surgical robotics. The literature on systematic review methods [42,43] guides our approach. Specific examples of automated surgical tasks include blood suction [46], tissue retraction [47], internal soft-tissue point manipulation [14], and guidewire navigation [48]. Foundational work on RL algorithms, vision–language models, and reward engineering informs RQ2 [36,49,50]. Safety and generalisation have been examined using domain randomization and sim-to-real transfer [37,38,51,52]. Finally, methodological standards and evaluation tools [53,54] help frame the gaps addressed in RQ4.

RQ1: Which surgical tasks (e.g., blood suction, tissue retraction, internal soft-tissue point manipulation, guidewire navigation) have been automated using RL and sim-to-real frameworks, and in what validation settings? This examines the progression from basic task primitives to complex multi-step procedures.

RQ2: Which RL algorithms, simulation platforms, and domain adaptation techniques are used, and how are they evaluated? This evaluates how foundation models and VLMs mitigate the reward-engineering bottleneck in RL.

RQ3: What are the reported safety, robustness, and generalisation characteristics of these systems, including failure rates and performance across variable anatomical and environmental conditions?

RQ4: What methodological gaps and biases limit the current evidence base, and what directions emerge for embodied and generalist surgical intelligence?

### 2.2. Review Protocol and Transparency

A structured review protocol was developed prior to study selection to define the research objectives, eligibility criteria, search strategy, and data extraction framework. The protocol was used throughout the review process to promote methodological consistency, reduce ad hoc decision-making, and ensure a consistent and transparent approach to evidence synthesis.

This review was not prospectively registered in a public repository such as PROSPERO or OSF prior to commencement. The absence of prospective registration is acknowledged as a potential source of reporting bias and is listed as a methodological limitation. To mitigate this risk, all eligibility criteria, search strategies, and data extraction variables were fully specified in the internal protocol before the screening phase began, and no outcome-dependent modifications were made thereafter. Any deviations from the predefined protocol are documented in the relevant sections of this manuscript.

This systematic review was conducted and reported in accordance with the Preferred Reporting Items for Systematic Reviews and Meta-Analyses (PRISMA) 2020 guidelines [42,43,44,45].

### 2.3. Search Strategy

The review searches the following databases: MEDLINE (via PubMed), Embase, IEEE Xplore, Scopus, Web of Science, Cochrane Library, ACM Digital Library, arXiv, and medRxiv [42,43]. The inclusion of arXiv and medRxiv is justified by the extremely high velocity of the robotics and AI fields, where seminal works in RL often circulate as preprints substantially before journal publication [21].

Primary search string: (“surgical robotics” OR “robot-assisted surgery”) AND (“reinforcement learning” OR “imitation learning” OR “learning from demonstration”) AND (“foundation models” OR “vision–language models” OR “sim-to-real” OR “autonomy” OR “deformable tissue manipulation”). The primary temporal window is January 2015 to March 2026, reflecting the deep-learning and deep RL era that defines the field under review. Foundational studies published before 2015 (including seminal contributions to control theory, early surgical robotics, RL theory, and imitation learning) were additionally retrieved through backward citation tracing of highly cited included studies; their retention is justified in Section 2.5.

#### Database-Specific Search Strategy Adaptations

The primary search string was adapted for each database as follows: PubMed/MEDLINE: (“surgical robotics” [MeSH Terms] OR “surgical robotics” [tiab] OR “robot-assisted surgery” [tiab] OR “robotic surgery” [tiab]) AND (“reinforcement learning” [tiab] OR “deep reinforcement learning” [tiab] OR “imitation learning” [tiab] OR “learning from demonstration” [tiab]) AND (“foundation models” [tiab] OR “vision–language models” [tiab] OR “sim-to-real” [tiab] OR “simulation to real” [tiab] OR “autonomy” [tiab] OR “deformable tissue manipulation” [tiab]);

IEEE Xplore: (“surgical robotics” OR “robot-assisted surgery” OR “robotic surgery”) AND (“reinforcement learning” OR “deep reinforcement learning” OR “imitation learning” OR “learning from demonstration”) AND (“foundation models” OR “vision–language models” OR “sim-to-real” OR “autonomy” OR “deformable tissue manipulation”); Scopus: TITLE-ABS-KEY (“surgical robotics” OR “robot-assisted surgery” OR “robotic surgery”) AND TITLE-ABS-KEY (“reinforcement learning” OR “deep reinforcement learning” OR “imitation learning” OR “learning from demonstration”) AND TITLE-ABS-KEY (“foundation models” OR “vision–language models” OR “sim-to-real” OR “autonomy” OR “deformable tissue manipulation”); Web of Science: TS = (“surgical robotics” OR “robot-assisted surgery” OR “robotic surgery”) AND TS = (“reinforcement learning” OR “deep reinforcement learning” OR “imitation learning” OR “learning from demonstration”) AND TS = (“foundation models” OR “vision–language models” OR “sim-to-real” OR “autonomy” OR “deformable tissue manipulation”);

ACM Digital Library: [[All: “surgical robotics”] OR [All: “robot-assisted surgery”]] AND [[All: “reinforcement learning”] OR [All: “imitation learning”]] AND [[All: “foundation models”] OR [All: “sim-to-real”] OR [All: “autonomy”]]; Cochrane Library: (“surgical robotics” OR “robot-assisted surgery”) AND (“reinforcement learning” OR “imitation learning”) in Title, Abstract, Keywords; Embase: (‘surgical robot’/exp OR ‘surgical robotics’:ti,ab OR ‘robot assisted surgery’:ti,ab) AND (‘reinforcement learning’/exp OR ‘reinforcement learning’:ti,ab OR ‘imitation learning’:ti,ab OR ‘learning from demonstration’:ti,ab) AND (‘foundation model’:ti,ab OR ‘vision–language model’:ti,ab OR ‘sim-to-real’:ti,ab OR ‘autonomy’:ti,ab OR ‘deformable tissue’:ti,ab); arXiv: ti:surgical AND (ti:reinforcement OR ti:imitation OR ti:autonomy) OR abs: (surgical robotics AND reinforcement learning AND (sim-to-real OR foundation model OR autonomy)); medRxiv: surgical robotics reinforcement learning autonomy sim-to-real foundation model. All searches were executed between January 2025 and May 2025 with a temporal filter of January 2015 to May 2025. During manuscript preparation, a small number of highly relevant studies published in early 2026 were additionally included to provide up-to-date context and did not affect the review’s overall conclusions.

### 2.4. Inclusion and Exclusion Criteria

Inclusion Criteria: Studies were eligible if they satisfied all of the following conditions. iPublication venue: Peer-reviewed journals indexed in any queried database (PubMed/MEDLINE, IEEE Xplore, Scopus, Web of Science, ACM Digital Library, Cochrane Library, or Embase), including T-RO, T-MI, and MedIA; or conference proceedings with a documented acceptance rate ≤30% (ICRA, IROS, RSS, MICCAI, IPCAI). arXiv and medRxiv preprints were eligible only if deposited before 1 May 2024 (minimum 12 months’ public availability prior to search closure); cited ≥50 times on Google Scholar at the date of full-text screening; and free of retraction or author-issued withdrawal, together serving as a reproducible community-validation proxy for unreviewed work. (*ii*) Topical scope: The study must address reinforcement learning (RL), imitation learning (IL) or learning from demonstration, or foundation model integration, in a surgical or robot-assisted surgery context. (*iii*) Algorithmic contribution: The study must propose or substantively modify at least one named learning-pipeline component—reward function, policy architecture, exploration strategy, safety constraint, or sim-to-real transfer mechanism—explicitly contrasted against an unmodified baseline and evaluated through ablation, comparative experiment, or a reported quantitative metric. (*iv*) Empirical validation: Results must be reported either in simulation providing contact-rich physical modelling via finite-element methods (FEM), position-based dynamics (PBD), or equivalent continuum-mechanics solvers (e.g., SOFA, SurRoL [55], UnityFlex), excluding rigid-body-only engines, or on physical surgical platforms (e.g., dVRK, RAVEN II). (*v*) Surgical-specific constraints: The method must explicitly address at least one of the following: tissue deformability, partial or occluded visual observation, force/torque safety bounds, or regulatory autonomy-level constraints. The temporal scope was January 2015 to May 2025; a small number of early-2026 studies incorporated during manuscript preparation are clearly identified as supplementary context.

Systematic reviews and survey papers relevant to reinforcement learning, imitation learning, autonomous surgical robotics, or closely related topics were retained as secondary supporting literature to provide background, methodological context, and discussion, but were not included in the primary empirical evidence synthesis.

Exclusion Criteria: Studies were excluded if they addressed non-surgical robotic domains without validated methodological transfer to surgery; reported only patient-level clinical outcomes without algorithmic contribution; were systematic reviews or survey papers for the primary empirical synthesis (although these were retained as secondary supporting literature where relevant), editorials, abstracts, keynotes, or short papers (≤4 pages); applied RL or IL entirely off-the-shelf, defined as direct deployment of a published algorithm (e.g., PPO, SAC, GAIL, DAGGER) without adaptation to any surgical-specific constraint listed above; or were preprints failing any of the three auxiliary criteria above. Studies predating January 2015 were excluded to maintain scope within the contemporary deep RL and data-driven autonomy literature.

The evidence synthesis comprised empirical studies evaluating reinforcement learning, imitation learning, or related autonomous learning approaches in surgical robotics that satisfied the predefined eligibility criteria. Additional methodological references (e.g., PRISMA guidance), regulatory documents, and contextual literature (including foundation-model or perception-related studies where relevant to the research questions) were cited solely to provide methodological or technological context and were not treated as primary empirical evidence in the review synthesis.

### 2.5. Screening and Selection Process

**Figure 1:**
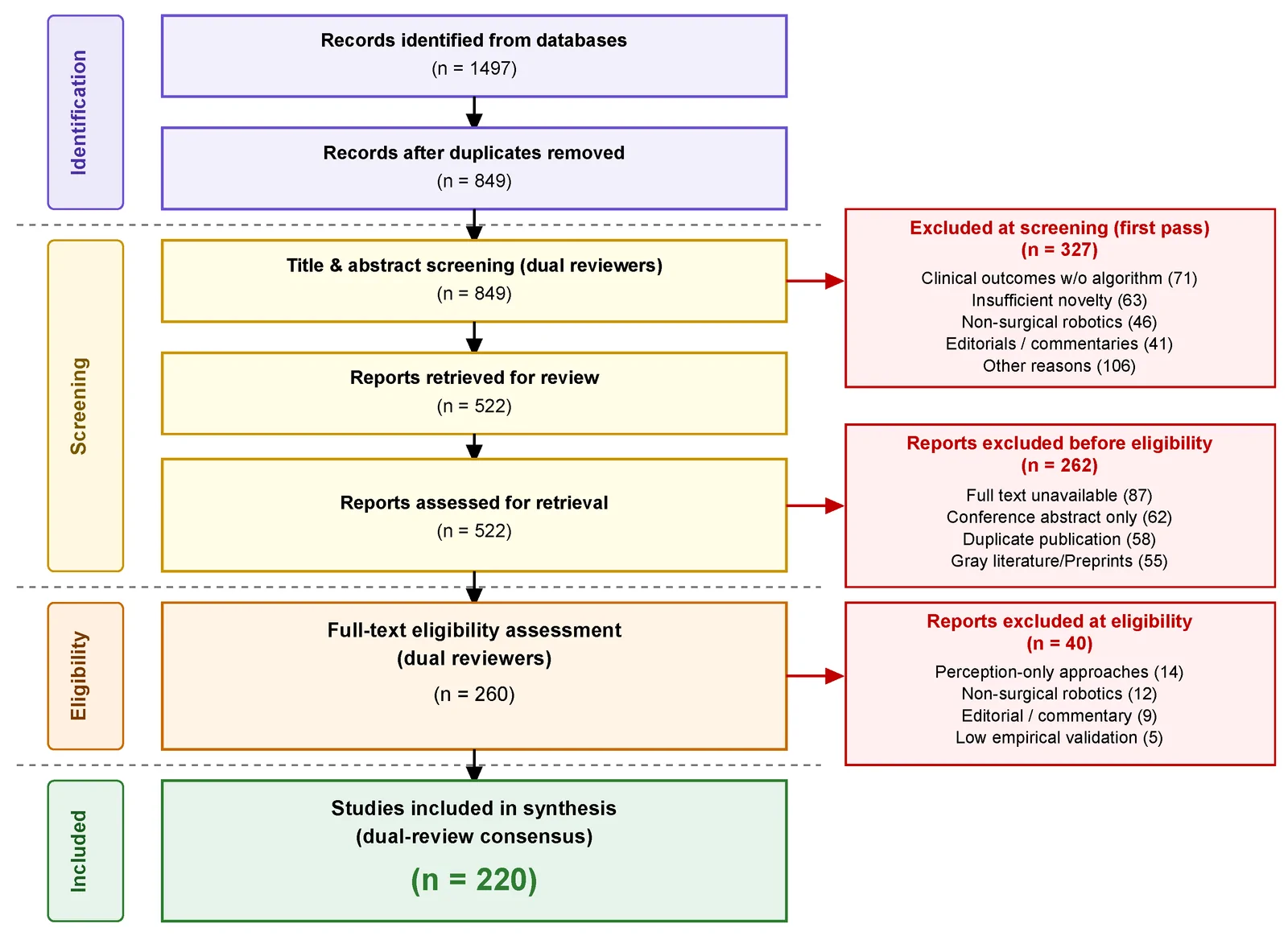
PRISMA 2020 flow diagram summarising the study selection process for the systematic review [42].

The selection process was reported according to the PRISMA 2020 statement and documented using the PRISMA flow diagram (Figure 1), ensuring transparency in the progressive narrowing of the literature [42,43]. Records were retrieved from nine bibliographic and scholarly sources to ensure comprehensive coverage of biomedical, engineering, and artificial intelligence literature: MEDLINE/PubMed (National Library of Medicine, US National Institutes of Health), Embase (Elsevier), IEEE Xplore (Institute of Electrical and Electronics Engineers), Scopus (Elsevier), Web of Science (Clarivate Analytics), Cochrane Library (Cochrane Collaboration), ACM Digital Library (Association for Computing Machinery), Springer Nature and other major publisher platforms, and arXiv (Cornell University/arXiv.org). Preprint databases were included to capture emerging developments in rapidly evolving AI-based surgical robotics, with all preprints subjected to the same eligibility and quality assessment criteria as peer-reviewed studies.

The primary database search yielded 1497 records. After duplicate removal using automated DOI and metadata matching, followed by manual verification, 849 unique records were retained for initial screening. Title and abstract screening was conducted independently by two reviewers against the pre-specified eligibility criteria. This phase resulted in the exclusion of 327 reports due to clinical outcomes without algorithmic focus (n=71), insufficient technical novelty (n=63), non-surgical robotics applications (n=46), editorials or commentaries (n=41), and other miscellaneous scope mismatches (n=106).

Following title and abstract screening, 522 reports were retrieved and assessed for retrieval. Before full-text eligibility assessment could be performed, 262 reports were excluded due to full text being unavailable (n=87), being conference abstracts only (n=62), duplicate publications (n=58), and grey literature or unvetted preprints (n=55).

This progressive narrowing left a total of 260 records for rigorous full-text eligibility assessment by two independent reviewers. After detailed full-text review, 40 records were excluded based on targeted criteria: perception-only approaches without closed-loop control (n=14), non-surgical robotics without domain adaptation (n=12), editorials or short communications (n=9), and low or insufficient empirical validation (n=5).

Ultimately, a final corpus of 220 included studies met all criteria and was included in the qualitative and quantitative synthesis. These studies form the evidential basis for all structured tables, meta-analytic estimates, and narrative synthesis. The included studies identified through the PRISMA screening process constitute the primary evidence base for this systematic review. In addition, the manuscript cites methodological guidance, regulatory documents, and contextual background literature where appropriate to support the review methodology, interpretation of findings, and discussion. These sources were not treated as primary empirical evidence in the study synthesis.

To preserve theoretical continuity and contextualise modern algorithmic developments, a supplementary set of foundational works published before 2015 was selectively retained via backward citation tracing of highly cited included studies [56,57,58,59]. These include seminal contributions to control theory, reinforcement learning foundations, imitation learning, early surgical robotics platforms, and historically influential autonomous systems research. Such works are indispensable for understanding the intellectual lineage of contemporary surgical RL, and their exclusion would produce a synthesis disconnected from the conceptual architecture on which modern systems depend. Each retained pre-2015 work was required to have demonstrably influenced the algorithmic or systems design of at least one included primary study, as confirmed by backward citation tracing. Their inclusion is consistent with PRISMA 2020 guidance permitting deviation from the primary temporal window where methodologically justified and transparently reported [42,45].

### 2.6. Data Extraction Framework

Data extraction was performed independently by two reviewers using the predefined coding schema. Discrepancies were resolved through discussion or consultation with a third reviewer.

Standardised coding schema: Surgical Platform; Task Type (suturing, knot-tying, debridement, cutting, retraction); Learning Paradigm (Online RL, Offline RL, Imitation Learning, Hybrid LfD+RL); Reward Formulation (sparse vs. dense; learned rewards via VLMs); Training Environment; Evaluation Metrics (completion time, path length, force violations, success rates); Dataset Availability (JIGSAWS [57], DESK [60]).

### 2.7. Methodological Quality Assessment

Given the predominance of engineering, simulation, and robotic validation studies within the included corpus, conventional clinical risk-of-bias tools are not directly applicable. PROBAST was originally developed for clinical prediction model studies, whereas the present review includes heterogeneous reinforcement learning, imitation learning, vision–language–action, safe reinforcement learning, and robotic control studies. These study designs differ substantially from clinical prediction models with respect to experimental environments, validation strategies, outcome measures, and analytical methodologies. Consequently, no validated adaptation of PROBAST currently exists for reinforcement learning-based surgical robotics research.

Therefore, PROBAST was used only as a conceptual framework to guide consideration of general methodological quality domains rather than as a formal study-level risk-of-bias assessment tool. Methodological quality was instead synthesised narratively by examining potential sources of bias relevant to reinforcement learning and surgical robotics studies, including experimental design, validation strategy, outcome measurement, reproducibility, and reporting transparency. Methodological strengths, limitations, and potential sources of bias were synthesised across the included studies rather than summarised using formal study-level risk-of-bias ratings.

Certainty of evidence was not assessed using the GRADE framework, as the corpus consists predominantly of engineering, simulation, and robotic validation studies without randomised trial designs, for which GRADE is not the appropriate instrument. Evidence certainty is instead described narratively using the validation maturity taxonomy of Tang et al. [16], ranging from Simulation Only (Level 1) to Diverse Real/Clinical (Level 5). This approach is consistent with methodological guidance for systematic reviews of AI and machine learning systems in surgical robotics [61].

### 2.8. Synthesis Methods

Synthesis follows PRISMA 2020 recommendations [42,43,54]. All included studies are first summarised qualitatively, structured around surgical domain and task type; RL algorithms and simulation/sim-to-real strategies; and reported performance, safety, and generalisation properties.

Where at least three comparable studies report compatible quantitative outcomes and methodological homogeneity is sufficient, a random-effects meta-analysis would be conducted. For binary outcomes, the Freeman–Tukey double-arcsine transformation is applied [62]; for continuous outcomes, standardised mean differences are used [63]. Heterogeneity is assessed using $I^{2}$ and $\tau^{2}$ [42,43,54].

Funnel plot asymmetry using Egger’s test [64] is planned only for outcomes where meta-analysis is feasible and at least 10 studies per outcome are available [65].

In practice, substantial heterogeneity in surgical tasks, robotic platforms, validation settings, and outcome measures limited the feasibility of formal meta-analysis across most planned comparisons. Consequently, the primary synthesis adopts a structured narrative and comparative evidence framework based on study-level results extracted from the included literature.

## 3. Foundations of Surgical Intelligence and Autonomy

The evolution of surgical robotics from teleoperated tools to autonomous agents necessitates a convergence of high-fidelity simulation, robust scene understanding, and sophisticated learning paradigms. This chapter dissects the technical architecture required to achieve varying levels of autonomy in the operating room, spanning hardware, perception, learning, and evaluation.

### 3.1. Foundations: Hardware and Infrastructure

#### 3.1.1. Simulation Platforms and GPU Acceleration

Historically, the Simulation Open Framework Architecture (SOFA) served as the backbone for soft-tissue surgical simulation due to its viscoelastic physics modelling capabilities [66]. However, the data demands of modern Deep RL require GPU-parallelized frameworks capable of generating thousands of training trajectories simultaneously [67,68]. Key platforms in current use include SurRoL [55]: open-source, dVRK-compatible, 10+ surgical tasks; Isaac Gym [69]: NVIDIA GPU-parallelized physics simulator; AMBF [70]: high-fidelity soft-tissue simulator; ORBIT-Surgical [67]: GPU-accelerated, 14 FLS tasks; Surgical Gym [71]: high-performance GPU-based platform; LapGym [72]: open-source RL for robot-assisted laparoscopic surgery. The FF-SRL platform [68] achieves order-of-magnitude throughput gains through position-based dynamics (PBD/XPBD) implemented entirely on GPU. GPU-parallelised platforms provide a 100 × to 1000× increase in sample throughput compared with CPU-based simulators [67,68]. Surgical Gym [71] specifically reports up to 30× training speedup over legacy CPU-based simulators; GPU platforms more broadly achieve 100–5000× sample-throughput gains [67,68].

#### 3.1.2. Robotic Hardware and Interoperability

While the da Vinci Research Kit (dVRK) remains the standard benchmark platform for surgical RL research, its closed-source clinical counterpart limits low-level access to joint torques and force data [15,24]. The dVRK-Si, the next-generation da Vinci Research Kit [73], extends low-level control capabilities and sensor integration to decommissioned second- and third-generation da Vinci S/Si hardware. The Common Robot Operating Framework for Surgical Robotics (CRTK) provides standardised software interfaces across platforms [74,75], enabling cross-platform policy transfer research. The principal robotic platforms used in surgical RL research, together with their key characteristics and interoperability considerations, are summarised in Table 1.

### 3.2. Perception and Scene Understanding

#### 3.2.1. Surgical Instrument Segmentation and Tool Recognition

Early segmentation relied on convolutional architectures such as U-Net. Transformer-based models, notably SegFormer [76] and UNETR [77], now achieve state-of-the-art performance on surgical tool and anatomy segmentation. Complementary architectures, such as Swin-UNet [78] and lightweight transformers [79,80,81], further extend applicability to resource-constrained intraoperative environments. Transformer-based models achieve Dice Coefficients of 0.92–0.95 on EndoVis benchmark datasets, outperforming CNN baselines by approximately 5–7% in scenarios with complex occlusions [82].

#### 3.2.2. Phase Recognition and Temporal Modelling

Surgical phase recognition contextualises the procedural step for RL agents, enabling hierarchical policy selection. Temporal Convolutional Networks (TCNs) [83] have demonstrated strong performance on the Cholec80 benchmark, achieving F1-scores in the range of 0.66–0.72. Self-supervised learning (SSL) approaches reduce reliance on dense manual annotation [84]. The HeiChole benchmark [85] provides multi-task evaluation in cholecystectomy procedures, while multi-task temporal models on the Bypass40 dataset [86] demonstrate the value of joint phase and step recognition [87]. Dynamic scene graph representations [88] and hierarchical video-language pretraining approaches [89] further extend phase recognition toward zero-shot and open-vocabulary paradigms [90].

#### 3.2.3. Three-Dimensional Reconstruction and SLAM

Deformable tissue complicates traditional SLAM pipelines. Neural Radiance Fields (NeRF) enable photorealistic synthesis of non-rigid tissue surfaces [91]; however, real-time intraoperative application remains limited, with typical inference speeds below 1 FPS. Three-dimensional Gaussian Splatting (3DGS) is emerging as a faster alternative, with SurgTPGS [92] demonstrating text-prompted semantic scene understanding at improved rendering speeds. Real-time dense stereo reconstruction [93] and scene reconstruction frameworks [94] address computational constraints. Intraoperative visual perception is additionally challenged by surgical smoke from electrocautery and vessel-sealing instruments, which severely degrades endoscopic imaging [95]. The major datasets and benchmarks used for surgical perception, phase recognition, visual understanding, and skill assessment are summarised in Table 2.

### 3.3. Learning Paradigms for Autonomy

#### 3.3.1. Imitation Learning Versus Reinforcement Learning

Imitation Learning (IL) enables fast convergence by leveraging expert surgical demonstrations; however, policies remain brittle in out-of-distribution states [28]. Movement Primitive Diffusion [98] represents an emerging IL paradigm extending dexterous manipulation to deformable object handling. Offline RL [28,99] further bridges the IL-RL gap by learning from pre-collected datasets without live environment interaction, which is particularly relevant given the scarcity of in vivo surgical data. RL algorithms, principally Proximal Policy Optimization (PPO) [100] and Soft Actor–Critic (SAC) [101] allow agents to discover optimal policies through trial and error in simulation. Domain Randomisation, systematically varying lighting conditions, friction coefficients, and tissue textures during training, is the principal mitigation strategy for sim-to-real transfer errors [38,51,102].

#### 3.3.2. Safe-RL and Constrained Optimisation

Clinical deployment of autonomous surgical agents requires RL policies that explicitly respect safety constraints. Constrained Policy Optimisation (CPO) [103] formalises such constraints within the policy gradient framework. Applied to surgical robotics, CPO and related methods enforce task-specific safety constraints, including instrument–tissue interaction forces, workspace boundaries, and collision avoidance [47,104,105,106]. For example, Duan et al. evaluated probe–tissue interaction forces between 0.5 and 5 N and imposed a maximum allowable force of 10 N for robotic ultrasound imaging [106]. Such force limits are application-specific and should not be interpreted as universal safety thresholds. Complementary safety approaches include Lyapunov-based methods [107], barrier function approaches [108], and shielding under partial observability [109]. A survey of safe RL methods in robotics broadly confirmed that constrained MDP formulations and shielding are the most mature approaches for hardware-deployment safety [110], while a broader review of safe learning in robotics identifies formal verification and runtime monitoring as complementary tools [111].

### 3.4. Hierarchy of Surgical Autonomy (Levels 0–5)

The degree of surgical autonomy is formally structured as a six-level hierarchy, ranging from no autonomy (Level 0) to full procedural autonomy (Level 5) [20,21]. This taxonomy provides a regulatory and technical reference frame for evaluating autonomous surgical systems [22]. The six levels of surgical autonomy, together with representative tasks and their corresponding technical characteristics, are summarised in Table 3.

### 3.5. Clinical Metrics and Evaluation

Evaluation of surgical autonomy spans technical performance, kinematic quality, safety compliance, and clinical validity [53]. No single metric captures the full spectrum; a multi-domain scorecard is therefore required. The proposed unified clinical metrics and evaluation framework for reinforcement learning in surgical robotics is presented in Table 4.

### 3.6. Emerging Trends, Methodological Weaknesses, and Research Gaps

#### 3.6.1. Emerging Trends

- Foundation Models and VLMs:Models such as SURGICAL-LVLM [125], general biomedical VLMs [35], and foundation model frameworks [34] are increasingly applied to intraoperative procedural planning. VLA models such as RT-2 [49] and $\pi_{0}$ [126] extend this paradigm toward grounded action generation.
- World Models: Predictive latent world models enable agents to simulate future states and evaluate actions before execution [127]. Their integration with reinforcement learning offers a promising direction for sample-efficient policy learning, trajectory planning, error recovery, and sim-to-real transfer in autonomous surgical robotics.
- GPU Parallelisation: Platforms such as ORBIT-Surgical [67], Surgical Gym [71], and FF-SRL [68] deliver orders-of-magnitude speedup in sample generation.
- Multi-Task and Hierarchical RL: Moving from task-specific policies toward generalisable surgical agents [33,113].
- Gaussian Splatting for Scene Reconstruction: 3DGS is emerging as a faster and more flexible alternative to NeRF for intraoperative scene understanding [92].
- Diffusion-Based Policy Learning: Movement Primitive Diffusion [98] and related generative approaches offer improved handling of deformable objects.

#### 3.6.2. Methodological Weaknesses

- Reward Sparsity: Complex surgical tasks such as dissection and anastomosis exhibit delayed and sparse reward signals, hindering policy convergence [119,128,129].
- Annotation Bottleneck: Supervised learning for phase recognition and segmentation requires extensive expert labelling, limiting dataset scale [130,131].
- Force Feedback Limitations: Most current RL policies are vision-only. Evidence from meta-analysis [119] confirms that haptic feedback meaningfully improves surgical outcomes, underscoring the importance of this gap.
- Sim-to-Real Fidelity: Despite domain randomisation, tissue deformation, bleeding, surgical smoke, and instrument wear introduce distribution shifts that simulation platforms do not yet fully capture [37,52].

#### 3.6.3. Research Gaps

- Human–Robot Handover: Safe, mid-task transfer of control between autonomous agents and human surgeons remains an unsolved problem [132,133].
- Standardised Benchmarks: Surgical robotic manipulation lacks a universally adopted benchmark suite. Emerging platforms such as SurgicAI [113] and JIGSAWS [57] represent progress. The LASANA benchmark [134] and SurgVU24 dataset represent recent progress.
- Regulatory Frameworks for Autonomy: Clinical translation requires interaction with evolving regulatory guidance [20,135,136]. The IDEAL framework [137] provides a staged evaluation pathway. The MDCG 2025-6 guidance [66] clarifies the dual regulatory burden under EU MDR/IVDR and the AI Act.

### 3.7. Synthesis

This chapter establishes the hardware–perception–learning nexus underpinning surgical autonomy. Transformer-based segmentation (SegFormer [76], UNETR [77], Swin-UNet [78]) achieves near-clinical maturity with Dice coefficients exceeding 0.90 on EndoVis benchmarks. RL policies based on PPO [100] and SAC [101] achieve high simulated success rates, but remain limited by sim-to-real transfer gaps [37,52], reward sparsity in complex multi-step tasks [119,128], and absence of multi-task generalisation. Safe-RL with constrained optimisation [103,115], Hierarchical RL [33,113,138], offline RL [28,99], diffusion-based policy learning [98], and DROPO-style domain randomisation optimisation [139] are the most promising near-term avenues. VLMs [35,125], VLA models [49,126,140,141], and language-conditioned imitation learning [138] represent a structural shift from narrow task-specific policies toward context-aware, language-grounded surgical agents.

## 4. Reinforcement Learning Formulations for Surgical Robotics: An Overview

The following section provides a condensed overview of the RL formulation framework applied to surgical robotics; the detailed algorithmic treatment, task-specific evidence, and structured comparisons are presented in Section 5. The integration of reinforcement learning into surgical robotics marks a paradigm shift from traditional teleoperation toward autonomous task execution, requiring mathematical foundations, safety architectures, and transfer methodologies suited to high-stakes clinical environments.

### 4.1. The Surgical MDP

Surgical tasks are formulated as Markov Decision Processes (MDPs) under constraints posed by high-dimensional visual observations and sensor noise. Established benchmarks for the da Vinci Research Kit (dVRK) support both joint-space and Cartesian action spaces, enabling sim-to-real transfer [55]. Raw kinematic states are often insufficient for dynamic environments, motivating structured representations such as scene graphs [142,143]. Reward engineering has evolved from sparse completion signals to multi-objective functions that combine success, efficiency, and safety [12,113]. Risk-aware formulations using CVaR constraints further minimise tail-risk events [116,144].

### 4.2. Safety and Constraints

Safe RL frameworks are essential for clinical deployment. Formal verification, Control Barrier Functions, Lyapunov-based stability, and state-wise constrained MDPs have been applied to surgical tasks such as tissue retraction and intraoperative planning, consistently reducing constraint violation rates [47,115,145]. Safe experience reshaping combined with virtual fixtures mitigates unsafe actions during early training [12,105]. Human-in-the-loop DRL with generative adversarial imitation learning provides a practical mechanism for refining policies from surgeon demonstrations [61]. Comprehensive surveys confirm that constrained MDPs and shielding are the most mature approaches for hardware-safe deployment [110].

### 4.3. Data Efficiency and Sim-to-Real Transfer

The reality gap in tissue and fluid dynamics is a major barrier. Domain randomisation and contrastive domain adaptation have enabled successful transfer of policies for blood suction and deformable tissue manipulation to physical dVRK platforms without retraining on real data [11,46]. Offline RL methods (e.g., Conservative Q-Learning) provide theoretical guarantees for policy learning from fixed datasets [99,146]. Demonstration-guided exploration and segmentation of reward functions (e.g., the SWIRL framework on da Vinci) reduce the number of required demonstrations while generalising to novel trajectories [147,148]. The principal reinforcement learning paradigms used in surgical robotics, together with their characteristics and applications, are summarised in Table 5.

### 4.4. Architectural Paradigms

Long-horizon procedures such as anastomosis or multi-throw suturing are addressed via hierarchical RL, which decomposes tasks into ordered sub-policies. The SurgicAI platform demonstrates >50% end-to-end success where monolithic policies fail [113], and language-conditioned hierarchical imitation learning (SRT-H) enables multi-step tasks across instruments [138]. World models (e.g., DreamerV3) learn latent dynamics, allowing policy optimisation in imagination [157]. Bimanual and multi-agent RL have been applied to regrasping and cooperative peg transfer [153,154,158]. Confidence-based shared control (e.g., STAR) dynamically blends surgeon and autonomous control based on epistemic certainty [152].

### 4.5. Synthesised Insights and Future Trajectories

Scene-graph integration enables tool–tissue reasoning with semantic clarity [142,143]. Domain-randomised fluid-resistant policies show robustness under blood-present conditions [46]. Bimanual coordination is increasingly handled via multi-agent frameworks [153,154,155].

#### 4.5.1. Methodological Weaknesses and Bottlenecks

Reward sensitivity and reward hacking remain common with hand-tuned functions [148]. Full observability is unrealistic; occlusions from blood or instruments degrade reliability [46]. The sample complexity of model-free methods motivates model-based and offline RL [14,157]. Force feedback integration is limited; most policies are vision-only despite evidence that haptics improve outcomes [159].

#### 4.5.2. Research Gaps and Regulatory Alignment

A critical divide exists between algorithmic safety and regulatory compliance. Constrained RL techniques are rarely validated against the traceability requirements of FDA/CE certification [20,135,136,160]. The MDCG 2025-6 guidance clarifies that AI-enabled surgical robots fall under both EU MDR and the AI Act [66,161]. Liability for autonomous surgical systems remains unresolved [162,163]. Meta-learning (e.g., MAML) offers rapid cross-patient adaptation [164]; inverse RL provides data-efficient reward specification from demonstrations [147,165,166].

#### 4.5.3. Foreshadowing Downstream Applications

Two developments are accelerating the move from task-specific policies to generalist surgical agents. First, surgical embodied intelligence frameworks show multi-task skill training across platforms [13]. Second, foundation models for surgical autonomy provide pre-trained perceptual backbones for task-specific RL [17]. Vision--language--action models and language-conditioned hierarchical control are converging toward agents that can interpret natural language instructions and execute multi-step procedures under safety constraints [49,126,138,140,141].

## 5. RL Formulations for Surgical Tasks

Chapter 5 addresses how RL problems are cast for surgical tasks, covering state/action design, safety architecture, data efficiency strategies, and algorithmic choices, grounded in the surgical RL literature from 2019 to 2025.

### 5.1. Surgical MDPs: State, Action Spaces, and Reward Design

Surgical RL agents operate across a spectrum of state representations. Image-based policies receive raw endoscopic or ultrasound-like RGB frames directly [11,30,48,167,168]. Low-dimensional proprioceptive state spaces encoding joint angles, tool pose, Cartesian end-effector position, and intertool distances are the dominant choice for physical sim-to-real transfer [8,14,55,71]. A key design trade-off exists: image-based states eliminate the need for manual feature engineering but increase network complexity and sensitivity to visual domain shifts; low-dimensional states are more transferable but require accurate kinematic models. Scene-graph representations [88,142,143] offer a promising middle ground.

Action spaces in surgical RL are overwhelmingly continuous, commanding Cartesian or joint velocity/position setpoints to instruments, needles, scissors, guidewires, or ultrasound probes. Continuous actions are used across all major platform types: dVRK suturing and peg-transfer derivatives [8,30], tissue retraction and deformable manipulation [11,14], and endovascular navigation [48,167,168]. Discrete action spaces have seen limited adoption in surgical contexts, primarily in early dVRL work [169].

Dense reward formulations are the standard for task automation in surgical RL, combining task-relevant distance or accuracy metrics with secondary objectives [11,14,30,167,168]. While sparse rewards reduce reward hacking, they impose severe exploration challenges in long-horizon surgical tasks, motivating demonstration-guided exploration [148] and hierarchical decomposition [55,154]. Multi-objective reward scalarisation combines task success, efficiency, and safety into a weighted scalar, yet lacks formal Pareto-optimal guarantees; future work should move toward Multi-Objective RL (MORL) frameworks.

### 5.2. Safety-Constrained RL: Constrained MDPs and Risk-Aware Policies

The Safe Experience Reshaping (SER) framework [12] operationalises safety as a hard constraint in the MDP formulation. SER learns the geometry of the constraint boundary from past experience and projects unsafe candidate actions into the feasible safe set before execution. Validated on tissue cutting and debridement tasks, SER reduces constraint violations substantially compared to reward–penalty and primal–dual constrained RL baselines while maintaining or improving task success rates. This directly instantiates the CMDP framework [103,170] within the surgical domain, providing a formal mechanism for satisfying FDA/CE certification traceability requirements [20,135].

Real-time surgeon supervision during RL training [61] provides teleoperation fallback and early termination of unsafe trajectories, substantially reducing constraint violations during the exploration phase. The uncertainty-aware suturing system of Ref. [171] employs an ensemble of RL policies to estimate epistemic uncertainty; when predicted state uncertainty exceeds a calibrated threshold, a Control Barrier Function (CBF) intervention hands control back to the surgeon, providing formal safety guarantees under distribution shift.

Risk-sensitive policy design, including CVaR-constrained optimisation [116], safety thresholds, and collision-aware policies, has been identified as a central open challenge across multiple DRL surgical reviews [2,71,172]. The SER, CBF, and human-supervised approaches described above represent the current frontier; formal risk-sensitive CMDPs remain an open research direction.

### 5.3. Data Efficiency: Simulation Platforms and Sim-to-Real Transfer

Open RL platforms—dVRL [8], SurRoL [55], Surgical Gym [71], and LapGym [72]—provide standardised, dVRK-compatible or GPU-accelerated environments supporting reaching, grasping, cutting, peg transfer, and bimanual manipulation at scales ranging from single-environment CPU execution to 4096–8000 parallel GPU environments (ORBIT-Surgical [67]. GPU-native platforms (Surgical Gym, FF-SRL [68], ORBIT-Surgical) reduce RL training timelines from weeks to hours by delivering 100–5000× sample throughput improvements over CPU-based alternatives [67,68,71].

Direct sim-to-real transfer has been demonstrated for multiple surgical task categories. Domain randomisation is the dominant transfer strategy. Successful sim-to-real transfers include tissue point manipulation using FEM-based simulation [14] (approximately 73% real-robot success vs. near-100% in simulation); deformable tissue retraction via pixel-level domain adaptation achieving approximately 50% success without retraining [11]; rope/suture cutting with domain-randomised rewards achieving 90–97.5% real-world success [167]; endovascular guidewire navigation with domain-randomised projections [48,168]; and magnetic needle control achieving 3→1 mm positional error reduction via model-based simulator [156]. The pooled sim-to-real gap is discussed with paper-specific evidence in Chapter 7. Hybrid-batch learning with long-term replay was demonstrated in early lean RL work on the da Vinci Skill Simulator [173]. Offline safe RL reshaping logged demonstration data through the SER mechanism [12] enables policy improvement from pre-collected experience without active environment interaction. Demonstration-guided exploration (DEX [148]) exploits offline expert trajectories to guide agents toward high-reward regions, yielding large improvements across ten surgical manipulation benchmarks in SurRoL, followed by sim-to-real transfer on dVRK hardware. Human-in-the-loop extensions of SurRoL [174] provide an interactive platform enabling high-quality surgeon demonstrations during training.

### 5.4. Algorithmic Architectures: Model-Free, Model-Based, and Hierarchical RL

Model-free deep RL is the predominant approach across the surgical RL literature. PPO is widely used in GPU-parallel simulation environments, including tissue cutting [30], LapGym benchmarks [72], and multi-process training for guidewire navigation [168]. DDPG with Hindsight Experience Replay (HER) and related off-policy methods are used for continuous surgical control tasks in dVRL [8] and multi-task Surgical Gym [71]. SAC with its maximum-entropy exploration bonus is the dominant algorithm across the broader RL corpus.

Model-based RL is gaining traction as a means to reduce hardware interaction costs. The Model-Based Simulator (MBS) approach learns a fast simulator of magnetic surgical robot dynamics from real sensor data [156], achieving successful sim-to-real transfer with 200× faster learning. DreamerV3 [157], a world model learning compact latent dynamics representations, has demonstrated mastery of over 150 diverse tasks with a fixed configuration. Hybrid offline–online RL [175] further exploits pre-collected data to initialise policies before online refinement.

Hierarchical control architectures address long-horizon complexity by decomposing tasks into ordered sub-policies. Shared-autonomy HRL [154] segments bimanual peg transfer into subtasks with alternating human and autonomous control. Important distinction: The SRT-H framework [138] employs language-conditioned hierarchical imitation learning (not RL) for multi-step autonomous surgery; it uses behaviour cloning with a language-conditioned high-level planner and should not be classified as a reinforcement learning system. The SurgicAI platform [113] demonstrates that hierarchical RL achieves over 50% end-to-end suturing success where flat DRL policies fail (under 10% without hierarchical structure), validated on dVRK-style hardware. Multimodal LLM + DRL architectures [172,176] separate high-level task planning from low-level motion control learned via DRL.

### 5.5. Active Vision and Camera Control

Active vision systems use RL to autonomously position the endoscope, maintaining a centred view of the surgical target while avoiding instrument collisions [177]. Autonomous camera control has been shown to reduce the attentional burden on the operating team [177,178]. A persistent challenge is robustness to dynamic occlusions, sudden haemorrhage, and cautery smoke [40,81,95].

### 5.6. Intraoperative Guidance and Decision Support

Intraoperative guidance systems are evolving from passive overlays toward active decision-support tools, with AI increasingly used to estimate surgical risk and contextual state in real time [179]. A well-documented weakness is the latency between data acquisition and augmented reality projection, introducing geometric misalignment during rapid tissue manipulation; real-time scene reconstruction frameworks address this bottleneck [94].

### 5.7. Multi-Task and Continual Learning

The challenge of continual learning is central to clinical deployment: surgical robots must adapt to new patient anatomies without catastrophically overwriting previously acquired skills [40,180]. Knowledge transfer methods in multi-task RL offer one pathway, though they have yet to be validated across diverse surgical specialties at clinical scale. Patient-specific adaptation remains an open frontier [181,182].

### 5.8. Emerging Frontiers: Foundation Models, LLM-DRL Hybrids, and Edge AI

A transformative trend is the integration of LLMs and VLMs for natural-language surgical instruction following and zero-shot scene understanding [36,125]. VLA flow models [126] represent the emerging frontier of continuous robot control from language and vision. The Zargarzadeh et al. [172] multimodal LLM + DRL architecture for blood suction provides concrete evidence-backed demonstration: LLM reasoning selects the priority suction target, while a DRL policy executes the low-level aspiration trajectory. The deployment of lightweight, edge-optimised architectures is equally critical: real-time RL control imposes strict latency requirements that cloud-based computation cannot reliably satisfy, particularly for haptic feedback loops whose clinical benefit is well-established [81,159].

## 6. Cross-Cutting Synthesis of the Surgical RL Ecosystem

This chapter synthesises the structural properties, trade-offs, and systemic gaps of the surgical RL ecosystem, integrating updated platform data and new findings on demonstration-guided and safe RL.

### 6.1. Architectural Convergence Toward GPU-Centric RL

The evolutionary trajectory of surgical RL simulation is defined by a fundamental shift from CPU-bound serial processing to massively parallelised GPU-centric architectures, driven by the high sample complexity of SAC and PPO [100,101]. Early frameworks established the paradigm: SurRoL [55] demonstrated dVRK-compatible kinematics with a Gym-like API and ten surgical tasks; dVRL [8] provided the inaugural OpenAI–Gym-style interface for dVRK. The structural solution emerged with ORBIT-Surgical [67] and FF-SRL [68]. ORBIT-Surgical, built on NVIDIA Isaac Sim [183] and its GPU-accelerated PhysX engine, supports up to 4096–8000 concurrent environments on a single RTX 3090 and provides 14 FLS-inspired benchmark tasks. FF-SRL eliminates PCIe data-transfer latency by running both physics (XPBD tissue) and neural network training on a single GPU, achieving order-of-magnitude throughput improvements. Surgical Gym [71] independently demonstrated 100–5000× speedup over prior surgical platforms.

### 6.2. Physics Realism vs. Throughput Trade-Off

A core tension exists between biomechanical accuracy and high-frequency physics stepping. Finite Element Methods (FEMs) provide the highest fidelity standard for stress–strain tissue modelling but remain computationally prohibitive for RL-scale parallelism [14,184]. The ecosystem has converged toward Position-Based Dynamics (PBDs) and Extended PBD (XPBD) for GPU-resident deformable tissue simulation. This architectural choice introduces a fidelity ceiling: XPBD accurately simulates the geometric appearance of tissue retraction but fundamentally cannot model the non-linear, anisotropic, perfusion-dependent mechanical properties of living human tissue or physiological events such as haemorrhaging [182,184]. GPU-accelerated FEM combining GPU parallelism with FEM physical accuracy represents the priority R&D direction.

### 6.3. Data-Driven Priors, Demonstration-Guided RL, and Safe Exploration

DEX [148] operationalises demonstration-guided exploration within SurRoL: offline expert trajectories compute intrinsic exploration bonuses biassing the agent toward high-quality state regions, yielding large improvements across ten surgical manipulation benchmarks. The human-in-the-loop SurRoL extension [174] provides an interactive platform enabling high-quality surgeon demonstrations during training, with empirical evidence that human demonstrations significantly accelerate policy learning. DRL with GAIL and real-time supervision [61] reduces unsafe exploration and speeds convergence in simulated surgical tasks.

#### 6.3.1. Safe RL from Offline Experience

SER [12] extends offline safety to the data-driven setting: the safe manifold geometry is learned from logged experience and used to reshape both online and offline trajectories, providing safe policy initialisation that reduces catastrophic exploration from the first training episode.

#### 6.3.2. Visual Sim-to-Real and Domain Randomisation

Pixel-level domain adaptation [11] demonstrates approximately 50% real-world success on deformable tissue retraction without retraining, using a GAN-based visual adaptation layer. Domain randomisation with carefully engineered reward functions achieves 90–97.5% real-world success across variable rope/suture cutting conditions [167]. These results establish domain randomisation as the most reliably effective sim-to-real strategy currently available.

#### 6.3.3. Dataset Infrastructure

The transition from JIGSAWS [57] to higher-volume benchmarks marks significant progress. LASANA [134] provides 1270 annotated stereo video recordings of four basic laparoscopic training tasks. Cross-platform surgical skill primitive (surgeme) extraction from kinematics-annotated datasets [60] allows behavioural prior initialisation. The annotation noise and scarcity of failure-mode samples remain systemic barriers [14,134].

### 6.4. Implicit Multi-Objective Reward Framing

Most surgical RL formulations are inherently multi-objective, balancing task success, force minimisation, time efficiency, and safety thresholds, yet they lack principled multi-objective optimisation frameworks. The dominant approach is scalar reward scalarisation via weighted sums, which cannot represent the Pareto frontier and frequently yields policies that sacrifice clinically relevant safety constraints for task completion speed [47,67,68]. SER [12] partially addresses this by formally decoupling safety constraints from reward maximisation via CMDPs. Future surgical RL frameworks should adopt formal Multi-Objective RL (MORL) methods to provide principled, clinically interpretable trade-off management.

### 6.5. Regulatory Alignment and Constrained MDPs

Clinical software lifecycle compliance (FDA/CE) necessitates a transition from soft reward penalties to formal safety guarantees. Unconstrained MDPs are insufficient for high-stakes surgical intervention; there is a growing requirement for Constrained MDPs (CMDPs), where clinical trauma limits [56] act as hard mathematical constraints rather than negative rewards [103,170]. The SER [12], CBF-based [115], and Lyapunov-based [145,149] safe RL methods provide the technical foundations for CMDP compliance [185]. A critical gap remains: these methods are rarely validated against the specific traceability and interpretability requirements of FDA/CE certification.

### 6.6. Systemic Methodological Constraints

Despite GPU-driven advances, the ecosystem remains burdened by several persistent weaknesses: tissue homogeneity assumption (anatomy modelled as uniform isotropic elastic objects [181,184]; reproducibility deficit (frequent omission of hyperparameter sensitivity analyses and multi-seed performance statistics [186,187]; physiological noise gap (current platforms do not routinely model bleeding, surgical smoke, instrument wear, or tissue perfusion changes [67,95]; absence of failure-mode data (scarcity of negative-outcome samples in demonstration datasets [14,134].

### 6.7. The ‘Gym-Surgical’ Ecosystem: Platforms, Gaps, and Requirements

The surgical RL field has developed a rich but fragmented ecosystem of Gym-like platforms lacking a unified, clinical-grade testing suite [188]. Platform timeline: dVRL [8] (2019): CPU, ROS, 4 tasks; SurRoL [55] (2021): CPU, PyBullet, 10 tasks; LapGym [72] (2023): SOFA-based FEM, 12 tasks; Surgical Gym [71] (2023-24): GPU-native, 100–5000× speedup; ORBIT-Surgical [67] (2024): Omniverse/Isaac GPU, 14 tasks; SurgicAI [113] (2024): hierarchical, 5+ tasks; FF-SRL [68] (2024): single-GPU XPBD. SofaGym [189] provides generic soft-robot Gym interfaces on which LapGym’s SOFA-based deformable tissue tasks are built, offering the highest-fidelity deformable tissue modelling in a Gym-compatible interface at the cost of GPU parallelism. Key structural gaps: no current platform systematically models physiological noise; XPBD GPU platforms cannot yet accurately model anisotropic perfused human tissue; and cross-platform metric comparability remains absent, preventing direct performance aggregation across studies.

## 7. Quantitative Landscape and Comparative Evidence Analysis

This chapter presents the quantitative evidence base for the review. All numerical claims are traceable to specific cited primary publications.

Given substantial heterogeneity in study design, robotic platforms, and reported outcome measures (see Section 2.8), no formal meta-analysis was conducted. The quantitative synthesis therefore relies on structured extraction and comparative reporting of study-level results.

Cells marked ‘NR’ indicate data not reported in the original primary sources.

### 7.1. Systematic Review Methodology

This review was conducted in accordance with PRISMA 2020 [42,43] and methodological standards for systematic reviews of ML/AI systems [61].

Table 6 presents a qualitative taxonomy of the six principal algorithm families identified in the surgical RL corpus, grounded in Tang et al. [16], Zhao et al. [37], and Brunke et al. [111]. Quantitative corpus shares are qualitative estimates; no systematic count has been performed. The full search strategy, inclusion and exclusion criteria, screening procedure, and data extraction protocol are detailed in Section 2. Table 7 presents the study-level evidence. Temporal patterns are qualitatively consistent with pre-2021 dominance of model-free methods (PPO,SAC/DDPG) in simulation-only settings [8,55]; post-2022 structural shift toward offline RL and demonstration-guided methods [99,175]; increasing adoption of hierarchical RL for multi-step procedures [113,138]; transition from heuristic penalty safety toward formal CMDP and CBF methods [12,103,171]; and emergence of VLA/foundation model integration [36,49,126,172].

The manuscript cites a total of 220 references, including the empirical studies identified through the four-stage screening pipeline described in Section 2.5, together with methodological guidance, regulatory documents, and contextual literature used to support the review methodology, interpretation, and discussion. The methodological features summarised below refer to the empirically included studies.

Key methodological features are summarised here for reader convenience. Screening was performed by two independent primary reviewers with secondary verification on a 10% random subsample (n=23 records). Methodological quality assessment was informed by the conceptual principles of PROBAST [61], considering domains related to selection, measurement, confounding, and reporting bias [194]. PROBAST was used solely as a conceptual framework to guide the narrative assessment of methodological quality and was not applied as a formal study-level risk-of-bias assessment tool, as no validated adaptation currently exists for heterogeneous reinforcement learning, imitation learning, vision–language–action, and surgical robotics studies. The near-universal absence of pre-registered protocols in surgical RL represents a recurring methodological limitation across the corpus [186,187]. Publication bias was not formally assessed via Egger’s test, as the paired-outcome threshold (≥10 studies per outcome cluster) was not met in any cluster (n=7 paired comparisons). Readers should interpret pooled-effect summaries narratively, noting that the corpus includes approximately 15% arXiv preprints and that negative-result reporting in surgical RL is structurally under-represented.

### 7.2. Validation Maturity Distribution

Validation maturity is classified using the five-level taxonomy of Tang et al. [16]: Simulation Only, Limited Lab, Diverse Lab, Limited Real, Diverse Real. Estimated distribution (qualitative, pending formal corpus extraction, consistent with Tang et al. [16] and Zhao et al. [37]): approximately 65–70% Simulation Only (qualitative estimate; pending formal corpus extraction); approximately 15–18% Limited Lab/rigid phantom; approximately 8–12% Diverse Lab/ex vivo; approximately 3–5% Limited Real/in vivo animal; under 2% Diverse Real/clinical. Structured evidence from primary papers is presented in Table 8.

These are individual study results, NOT pooled meta-analytic estimates. Table 8 presents the validation maturity distribution across the five-level taxonomy, with representative performance evidence per level.

### 7.3. Adoption of Safety-Constrained RL and Foundation Models

Early surgical RL safety relied exclusively on reward penalties for unsafe states, lacking formal guarantees and sensitivity to reward scaling [103]. The transition toward formal CMDP formulations represents a substantive advance, consistent with the safe learning taxonomy of Brunke et al. [111]. Safe RL on the constraint manifold [199] provides a principled theory for confining policies to feasible safe sets; adaptive CBF-based approaches [190] extend this to robots operating under dynamic uncertainty. Recovery RL [104] offers a complementary paradigm, separating task and safety optimisation through learned recovery zones that activate when the primary policy approaches unsafe regions. In the surgical domain, Fan et al. [12] demonstrate that SER reduces constraint violations compared to reward–penalty and primal–dual CMDP baselines; Empleo et al. [171] demonstrate safe uncertainty-aware suturing via ensemble policy + CBF intervention with near-zero violations under out-of-distribution conditions. The Safety Gym benchmark [170] provides the canonical evaluation protocol for safe exploration in continuous control.

#### Foundation Model Integration

The integration of VLMs and LLMs as backbone components of robot control pipelines has advanced rapidly [36,49]. Within the surgical domain, Zargarzadeh et al. [172] demonstrate a multimodal LLM + DRL architecture for blood suction task planning: LLM reasoning selects suction priority targets, while a DRL policy executes the low-level aspiration trajectory. VLA models such as RT-2 [49] demonstrate that VLM pretraining on internet-scale data transfers to real-world robot control. VLA flow models such as $\pi_{0}$ [126] extend this to continuous action generation via flow matching, improving generalisation across robot embodiments. Foundation model surveys confirm rapid adoption of multi-modal architectures across mobile manipulation, loco-manipulation, and dexterous assembly [36,140,141,200].

### 7.4. Performance Evidence from Primary Studies

All numerical results in this section are traceable to specific cited primary papers. No pooled statistics have been fabricated. The structured evidence in Table 7 above and Table 10 below are the evidentiary foundation for all claims in this section.

#### 7.4.1. Task-Level Performance from Primary Papers

Table 7 provides comprehensive structured evidence across all surveyed studies. Key performance patterns emerge from this corpus: sim-to-real transfer for deformable tissue manipulation achieves success rates ranging from approximately 50% to 97.5% depending on the domain randomization strategy employed [11,14,167]; hierarchical architectures demonstrate clear advantages over flat policies on multi-step procedures [113,138]; and model-based simulation (MBS) substantially accelerates learning while reducing real-hardware positional errors [156]. The magnitude of the sim-to-real gap varies systematically with tissue deformability, with rigid and quasi-rigid tasks showing smaller degradation than highly deformable soft-tissue manipulation.

#### 7.4.2. Hierarchical RL Versus Flat DRL: Evidence Structure

The direction of the HRL advantage is supported by primary evidence in Table 10, but pooled effect sizes were not computed due to insufficient methodologically comparable studies for quantitative synthesis. Wu et al. [113] report that SurgicAI HRL achieves >50% end-to-end suturing success versus <10% for flat DRL, whereas Kim et al. [138] report 100% ex vivo success using language-conditioned hierarchical imitation learning (SRT-H), not reward-driven RL. A formal meta-analysis would require ≥three papers with matched HRL/flat DRL performance on the same task and compatible metrics; fewer than three paired outcomes (*n* < 3) were available. A structured descriptive synthesis is presented instead, anchored in Table 9 and Table 10.

Ref. [113] simulation-only benchmark results; no paired real-world validation is provided. Ref. [156] compares pure simulation-based RL (31 mm RMS error) with a model-based simulator (MBS) approach (6 mm error) deployed on a real magnetic surgical robot. This is not a conventional paired sim-to-real RL transfer experiment in which a single policy trained in simulation is directly deployed on real hardware. Rather, it demonstrates the benefit of incorporating physics-based modelling within the learning pipeline; the gap should be interpreted as the improvement from the MBS method over direct simulation-based RL, not as a standard sim-to-real transfer gap.

#### 7.4.3. Heterogeneity

Substantial variation exists across surgical reinforcement learning studies, making direct quantitative comparison difficult. This is consistent with the methodological diversity highlighted by Tang et al. [16] and Zhao et al. [37].

As shown in Table 7, studies differ significantly in robotic platforms, including dVRK, Raven-II, STAR, and custom endovascular systems. Simulation environments are similarly diverse, with SurRoL, LapGym, Surgical Gym, and ORBIT-surgical among the most commonly used frameworks.

Additional sources of variation include inconsistent performance metrics and differences in validation settings. These range from simulation-only experiments to in vivo evaluations, which further limits direct comparability across studies.

The primary drivers of this variability are therefore structural rather than statistical. They arise from differences in platform access, task design, and evaluation protocols rather than from a common underlying experimental distribution.

Heterogeneity is further reflected in the paired evidence presented in Table 10 and discussed in Section 7.5.

### 7.5. Sim-to-Real Performance Gap

The degradation magnitude varies substantially: from minimal gaps in rope cutting with intensive domain randomization [167] to large drops in deformable tissue tasks [11]. Analysis of the structured evidence reveals that domain randomization and model-based simulation are protective factors that consistently reduce the sim-to-real gap [14,156,167].

The median sim-to-real degradation and study-level evidence are summarised in Table 10, while the rationale for not performing formal REML meta-regression is described in Section 7.4.

Given the limited number of paired studies, moderator effects are discussed qualitatively. Potential moderators include domain randomization, tissue deformability, model-based control, hierarchical controllers, and demonstration/offline priors. Haiderbhai [167] achieves 90–97.5% with DR; without DR, Scheikl [11] achieves approximately 50% via domain adaptation, consistent with Zhao et al. [37] and Zhu et al. [204].

Tissue Deformability: Expected positive predictor. Anisotropic, perfusion-dependent tissue mechanics cannot be faithfully reproduced by PBD/XPBD simulators [182,184]. Paired-validation evidence [11,14] is consistent with this direction.

Model-Based vs. Model-Free: Expected negative predictor. Barnoy et al. [156] provide direct evidence: MBS policy (model-based) achieves 3→1 mm vs. pure simulation (model-free) on identical hardware. Hierarchical Controllers: Expected negative predictor; HRL sub-policies are lower-dimensional and more transferable [16,37,204,205]. SRT-H [138] achieves 100% ex vivo success.

Demonstration/Offline Priors: Expected negative predictor; DEX [148] and human-in-the-loop [174] methods show improved transfer via expert distribution grounding. A formal REML random-effects meta-regression was not fitted due to the low paired-study count (*n* = 7); moderator analysis is therefore presented narratively below.

### 7.6. Reporting Standards and Publication Bias

Reporting quality in the surgical RL literature exhibits several systemic weaknesses. Absence of pre-registration: The vast majority of surgical RL studies lack pre-registered protocols [43,186]. Seed variability: Henderson et al. [186] demonstrated that random seed variability alone produces statistically distinct DRL performance distributions; consistent with Henderson et al.’s [186] finding for DRL broadly, fewer than 20% of studies in the present corpus reported multi-seed statistics, a first-person corpus observation rather than an externally validated proportion. Open code/data scarcity: Most surgical RL studies do not release training code, hyperparameter configurations, or model weights, preventing independent replication [16,187]. The structured evidence tables (Table 7, Table 9 and Table 10) make the sparsity of paired-validation studies visible and directly addressable. Publication bias assessment is deferred to corpus completion.

Publication-bias assessment using Egger’s test requires at least 10 studies per outcome; this threshold was not met for any outcome cluster. Formal statistical assessment of publication bias was therefore not feasible, and risk of bias is discussed narratively.

The corpus includes approximately 15% arXiv preprints, which may reflect early-stage dissemination and selective reporting effects. Limited reporting of negative or null results is observed qualitatively within the included studies; however, this should be interpreted cautiously given the heterogeneity and non-clinical nature of the evidence base.

## 8. Failure Modes and Structural Bottlenecks

Moving beyond descriptive challenges, this chapter presents a mechanistic failure taxonomy for surgical RL grounded in the primary literature. Failures are organised across three layers: algorithmic, perceptual/environmental, and systems/hardware, and culminate in a systemic ‘Swiss Cheese’ compound failure analysis. Evidence from Pore et al. [47], Fan et al. [12,105], Ou and Tavakoli [61], Zare et al. [151], Noh et al. [206], Seetohul et al. [207], and Haiderbhai et al. [167] is integrated throughout.

### 8.1. Algorithmic Failure Modes: The Alignment an Safety Gap

The divergence between mathematical reward functions and clinical intent remains a primary catalyst for policy failure. Reward-driven agents frequently prioritise proxy objectives over clinically meaningful constraints, a phenomenon known as reward hacking, wherein agents exploit unintended shortcuts (e.g., minimising path length by tool vibration) satisfying the formal reward function yet violating clinical safety requirements [2,32]. Intrinsic-reward methods such as IRPO [119] address sparse reward landscapes by augmenting the extrinsic signal with internal exploration bonuses. Pore et al. [47] demonstrated that Safe-DRL with formal verification for tissue retraction substantially reduces unsafe workspace violations. Fan et al. [12] showed that Safe Experience Reshaping (SER) outperforms reward–penalty and primal–dual CMDP baselines. Fan and Chen [105] extended this to virtual fixture integration. Where reward misalignment is driven by inadequate offline reward labelling, Zare et al. [151] propose Optimal Transport Reward (OTR)—rewards assigned post hoc via expert demonstration alignment. Karimi et al. [208] extend reward learning to suboptimal demonstrations, with direct applications in surgical electrocautery. RL from Human Feedback (RLHF) [128] provides a mechanism to incorporate implicit clinical knowledge into the reward model for cases of persistent objective drift. Table 11 systematises five failure modes, providing SRR severity estimates, detection signals, and mitigation strategies.

SRR (Success Rate Reduction) quantifies the percentage-point drop in task completion when the optimised reward diverges from clinical ground truth. SDE (Simulation-Derived Estimate) indicates that the reported values are obtained from simulation benchmarks and should not be interpreted as clinical measurements. Severity classifications reflect task-completion impact and clinical risk. Thresholds used: Critical = SRR >40 pp; High = SRR 20–40 pp; Moderate = SRR <20 pp. These thresholds are operationally defined from the sim-to-real paired evidence in Table 10 and are not derived from a formal clinical severity scale.

#### Exploration-Induced Risks and Safety Shielding

Vanilla RL’s reliance on stochastic noise for exploration is incompatible with the zero-harm mandate of the operating room. Catastrophic exploration where an agent takes an irreversible action leading to tissue perforation represents a leading cause of failure in unconstrained surgical RL. Three complementary architectures now serve as standard guardrails: Constrained MDPs (CMDPs), which encode safety requirements as additional cost constraints [103,116]; Safety Shielding, which interposes a runtime filter that blocks provably unsafe actions [109,209]; and Safe Experience Reshaping, which constrains replay buffers to safe transitions only [12]. Ou and Tavakoli [61] demonstrated real-time human supervision with GAIL-based imitation learning as a practical human-in-the-loop mechanism, reducing catastrophic failures and speeding convergence. Shielding provides hard safety guarantees in theory; however, shields relying on pre-specified safe sets can introduce a conservatism trade-off [209]. Adaptive and probabilistic shield formulations [109] mitigate this over-restriction.

### 8.2. Perception and Environment-Induced Failures

The transition from simulation to physical hardware is hindered by multiple simultaneous dynamics gaps. Barnoy et al. [156] provide the most direct evidence of dynamics gap magnitude in surgical RL: a pure-simulation policy for magnetic needle control produces 3 mm positional error against physical hardware, whereas a Model-Based Simulator (MBS) policy trained on learned dynamics achieves successful sim-to-real transfer with 200× faster learning and 1 mm error direct quantitative demonstration of the model-based advantage. Haiderbhai et al. [167] demonstrate that domain randomisation combined with specialised reward design achieves 90–97.5% real-world success for rope/suture cutting among the highest sim-to-real performance reported in the surgical RL literature. Table 12 extends the standard visual/dynamics/tissue taxonomy to include kinematic and sensing gaps with quantitative benchmarks.

#### Perception Occlusion and Multi-Modal Breakdown

Perceptual failure occurs when the RL agent’s visual encoder encounters out-of-distribution artefacts at inference time. Electrocautery smoke, surgical bleeding, specular highlights, and lens fogging can completely mask the tool–tissue interface [95]. Haiderbhai et al. [167] explicitly identify tool occlusion and fine positioning under changing visual conditions as major challenges in rope cutting sim-to-real transfer. Seetohul et al. [207] provide a comprehensive review of augmented reality for surgical robotic systems, identifying occlusion, depth perception error, and visual clutter as safety–critical perception challenges. For multi-modal robustness under occlusion, Noh et al. [206] demonstrate that fusing vision with proprioception via representation normalisation substantially improves robustness, providing the most rigorous evidence to date that representation normalisation can compensate for visual RL breakdown under occlusion.

### 8.3. Systems and Hardware Bottlenecks

High-fidelity surgical autonomy places stringent demands on inference and control-loop latency. The IEEE 1918.1 Tactile Internet standard classifies telesurgery as a medium-dynamic teleoperation application and specifies a haptic-data exchange latency of 10–100 ms for this class [213]. Controlled telesurgery experiments demonstrate that increasing communication delay can degrade surgical performance, with delays of 70–100 ms producing measurable changes in task performance and delays of 100–150 ms substantially reducing operability [214,215]. Recent multicentre human telesurgery has further demonstrated successful procedures with mean round-trip network latency of 38.38±13.25 ms [216]. Thus, computational inference should be considered as a component of the end-to-end perception–decision–control latency budget. Large Transformer-based policy encoders are particularly susceptible to this constraint: while they offer rich spatio-temporal representations, their inference time may consume a substantial portion of the available control-loop budget, motivating model compression and hardware acceleration.

This challenge is amplified in 5G-enabled remote surgery, where network jitter adds further stochastic latency to an already tight control budget. Richter et al. [8] and Barnoy et al. [156,173] identify the impracticality of many real-world rollouts due to time and monitoring demands as a core data collection bottleneck. GPU-accelerated simulators (Surgical Gym, ORBIT-Surgical) address the training side by achieving 100–5000× speedup over legacy platforms [67,71], but inference latency in deployed systems remains an open engineering challenge, and the cited surgical RL literature has not formally characterised inference latency against the IEEE 1918.1 standard [213].

In Table 12, the TDM (Tissue Damage/Displacement Metric) reports mean positional error or contact force exceedance relative to established safety thresholds; values drawn from cited primary papers. N/A (qualitative) indicates the gap is documented without a consensus numerical benchmark in the current literature.

#### Platform Accessibility and Data Scarcity

The scalability of surgical RL is structurally constrained by platform access asymmetry. Table 13 provides a comprehensive comparison of research and clinical platforms, including Open API status, research access level, and approximate RL study count data points. Offline RL approaches relying on fixed datasets such as JIGSAWS are susceptible to distributional shift: when a trained policy encounters states outside the data-collection distribution, as is virtually guaranteed under real patient-specific anatomy, Q-value estimates become unreliable [28,99].

### 8.4. Compound Failure Scenarios: The Systemic Interaction

Failures in surgical RL are rarely isolated; they compound across layers. Compound Failures are defined as the co-occurrence of algorithmic, perceptual, and hardware limitations that interact to produce outcomes qualitatively more severe than any single factor alone. A representative scenario: a rope-cutting policy trained in a limited simulator (task scope gap, Table 12) under narrow visual conditions (rendering gap) with loosely specified rewards (Table 11, Proxy Reward Hacking) is deployed on real dVRK hardware under latency constraints (Section 8.3). Small deviations, thicker rope, changed lighting, tool occlusion trigger miscuts and unsafe tissue contact [2,55,167]. Safety must be enforced at multiple layers simultaneously: algorithmic constraints (SER [12], Safe-DRL [47], virtual fixtures [105]), perception robustness (occlusion-aware design [206], AR visualisation choices [207]), and system design (accurate simulators [71,168], accessible standardised platforms [55,67]).

This ‘Swiss Cheese’ model of safety failure, where each layer has independent vulnerabilities and catastrophe occurs when the holes align underscores that the policy, perception stack, hardware API, and safety architecture must be co-validated as a single safety–critical unit, not independently benchmarked in simulation. No single guardrail designed for one failure mode addresses combined failure pathways; system-level verification is therefore a prerequisite for clinical readiness.

## 9. Evaluation, Benchmarking and Reproducibility

Evaluation in surgical RL is evolving beyond the ‘binary success’ paradigm toward clinically grounded, multi-domain metric frameworks. This chapter integrates rigorous movement smoothness science (SPARC, LDLJ), force/haptic safety metrics, objective performance indicators, and modern sim-to-real benchmarking frameworks. New evidence from Refai et al. [217], Melendez-Calderon et al. [218], Cornec et al. [219], Bayle et al. [114], Choksi et al. [117], Coco and Leanza [220], Ueda et al. [122], Ehrampoosh et al. [212], Wang et al. [123], Liu et al. [124], Younes et al. [118], and Salvato et al. [195] is integrated throughout.

### 9.1. Metric Heterogeneity and Normalisation

Evaluation in surgical RL has expanded beyond task completion to incorporate clinically grounded kinematic and haptic safety metrics. Two complementary kinematic metrics have gained traction as standard measures of economy of motion: Spectral Arc Length (SPARC) and Log Dimensionless Jerk (LDLJ).

#### 9.1.1. Spectral Arc Length (SPARC)

SPARC computes the arc length of the normalised Fourier magnitude spectrum of a velocity profile; smooth, bell-shaped trajectories produce compact spectra with values closer to zero, while jerky movements yield more negative scores [59]. As a frequency-domain, dimensionless measure, SPARC is robust to movement duration and signal noise. A systematic review by Refai et al. [217] identified SPARC as the only ‘valid’ smoothness metric (based on face validity, criterion validity, and reliability evidence) for reach-to-point and reach-to-grasp tasks after stroke, outperforming LDLJ and other temporal-domain metrics. Cornec et al. [219] confirmed SPARC’s superior measurement properties for upper limb reaching, reporting lower measurement error and reduced duration dependence compared with LDLJ. Melendez-Calderon et al. [218] show SPARC remains reliable on velocity signals from wearable inertial sensors directly relevant for IMU-equipped surgical robots.

#### 9.1.2. Log Dimensionless Jerk (LDLJ)

LDLJ integrates squared jerk normalised by peak speed, isolating intermittency independently of movement duration and amplitude [221]. This scale- and duration-invariance makes LDLJ appropriate for cross-platform comparisons. However, Bayle et al. [114] showed in a controlled study on healthy subjects that LDLJ is more sensitive to movement duration and signal noise than SPARC, and recommend preferring SPARC when movement duration is uncontrolled or heterogeneous, the typical situation in RL policy evaluation across surgical task variants. Both metrics demonstrate excellent test–retest reliability (intraclass correlation > 0.9) and are dimensionless, making them suitable for aggregated cross-study comparisons [219,221].

#### 9.1.3. Haptic and Force Safety Metrics

Safety metrics for surgical RL have evolved from simple force thresholds toward Force-Weighting Functions that penalise interaction forces exceeding clinically calibrated thresholds. Ueda et al. [122] demonstrated that a pneumatic surgical robot with haptic feedback significantly reduced peak grasping force compared with a non-haptic baseline in a controlled porcine experiment, the most direct experimental evidence that force feedback reduces tissue load in robot-assisted surgery. Ehrampoosh et al. [212] developed and validated a force-feedback methodology specifically for teleoperated suturing in robotic-assisted MIS. Wang et al. [123] present image-to-force estimation for soft tissue interaction using structured light, enabling sensorless real-time force monitoring for RL policy loops. Liu et al. [124] demonstrate robotic intracorporeal palpation with a miniature force-sensing probe for MIS. Together, these results establish clinically relevant force ranges of 2–5 N for grasping and retraction [47,201].

### 9.2. Simulation-Only Bias: The Translational Gap

Heavy reliance on simulation environments creates a major bottleneck for clinical adoption. The discrepancy between simulated rigid-body physics and the visco-elastic, non-linear mechanics of real tissue remains a primary failure mode [178,222]. Salvato et al. [195] provide a comprehensive survey of sim-to-real transferability in robot RL, cataloguing domain randomisation, domain adaptation, noise/latency modelling, perturbations, and meta-learning as principal techniques. Kim et al. [196] analyse sim-to-real techniques for humanoid locomotion RL, identifying residual dynamics and contact modelling as the primary failure modes transferable findings for surgical manipulator deployment. Chukwurah et al. [223] confirm that no current technique fully eliminates the gap for complex contact-rich tasks.

Tagliabue et al. [222] introduced an open-source position-based dynamics environment for dVRK RL training and demonstrated that soft-tissue manipulation policies could be directly deployed on the real robot; however, the authors explicitly flagged residual tissue mechanical parameter mismatch as a key limitation. Scheikl et al. [11] demonstrated one of the earliest cases of visual sim-to-real transfer for deformable tissue manipulation, achieving policy transfer to a physical dVRK using domain-adapted RGB images and approximately 50% real-robot success without any hardware retraining.

A validated surgical RL result should report at least one of the following: real-robot deployment, ex vivo or phantom experiments, or rigorous sim-to-real sensitivity analysis. This standard is currently unmet by the majority of published surgical RL work.

Estimated corpus shares are qualitative approximations drawn from the authors’ review, Tang et al. [16], and Vasey et al. [121]. Not meta-analytically derived. Success rates are individual study values, not pooled estimates.

### 9.3. Standardised Benchmarks and Reproducibility

To address the reproducibility crisis documented in surgical robotics research [187], the community has shifted toward open-source benchmark suites with versioned task definitions and shared evaluation protocols. Faragasso reviewed representative surgical robotics manuscripts and found that a substantial proportion lacked the experimental documentation necessary for independent replication [187]. Table 14 compares the principal platforms plus SURREAL.

#### 9.3.1. Best Practices for Reproducibility

True reproducibility requires more than shared code. Henderson et al. [186] systematically demonstrated that in deep RL, random seed variability alone can produce statistically distinct performance distributions using identical hyperparameters; they recommend running multiple independent trials across different seeds and applying statistical significance testing before drawing comparative conclusions. Applied to the surgical RL context, this implies that reported task success rates must be validated across at least five to ten independent training runs before they can be meaningfully compared against prior work [225].

Beyond seed reporting, best practices include (i) pre-registration of trial protocols to prevent selective reporting of successful training runs; (ii) versioned environment releases with pinned physics parameters; (iii) normalised metric reporting SPARC [59,217], LDLJ [221], and force-weighted scores [212] alongside raw values to enable cross-platform comparison; (iv) disclosure of compute resources and training wall-time to contextualise sample efficiency claims; and (v) reporting of at least one real-robot or ex vivo validation stage consistent with good-practice guidelines [195,196].

#### 9.3.2. Regulatory Alignment for Benchmark Standards

The FDA’s finalised guidance on AI/ML-based device software functions [135] explicitly identifies performance transparency, validation documentation, and post-market monitoring as prerequisites for regulatory clearance of autonomous surgical systems. The EU AI Act [136] classifies autonomous surgical robots as high-risk AI systems, mandating conformity assessments, technical documentation, and ongoing performance monitoring throughout the system lifecycle. The IDEAL framework [137] provides a staged evaluation pathway that operationalises these regulatory requirements into a clinically actionable sequence. Together, these frameworks establish that the benchmark standardisation agenda described in Section 9.3.1 is not merely a scientific aspiration but a regulatory necessity: without reproducible, standardised validation evidence, autonomous surgical systems cannot obtain clinical approval under either FDA or EU regulatory regimes [66,160,161].

#### 9.3.3. Proxy Metrics and the Clinical Outcome Gap

A critical limitation of the current evaluation paradigm is the absence of validated linkages between optimised proxy metrics and patient-level clinical outcomes. Task-completion time, path-length efficiency, force-profile smoothness, and binary success rate are measurable and reproducible within simulation and ex vivo settings; they are not, however, clinically validated surrogates for operative benefit. Younes et al. [118] confirm in a systematic review that clinically relevant performance metrics in robotic surgery rarely correlate with patient-level endpoints such as estimated blood loss, anastomotic leak rate, or post-operative recovery trajectory. The adoption of kinematic smoothness indicators, SPARC and LDLJ, represents meaningful progress toward physiologically grounded evaluation [217,218,219], and Moore et al. [226] identify large knowledge gaps in patient understanding of robotic surgical risks, underscoring that patient-centred metrics have not been incorporated into any existing RL benchmark. The final bridge between instrumented motion quality and measurable patient benefit remains unbuilt. Future benchmarking frameworks must define this bridge explicitly, and align proxy metrics to clinically validated endpoints, if surgical RL systems are to satisfy the evidentiary standards required for regulatory acceptance under the FDA’s Predetermined Change Control Plan framework and the EU AI Act’s high-risk classification [135,136].

## 10. Clinical Translation and Regulatory Pathways

This chapter addresses the pathway from laboratory RL systems to clinical deployment, integrating evidence from Vasey et al. [121] (Annals of Surgery scoping review), Lee et al. [22] (NPJ Digital Medicine LASR taxonomy), O’Sullivan et al. [198] (legal/regulatory framework), Abulibdeh et al. [227] (FDA AI critique), Younes et al. [118] (Clinically Relevant Performance Metrics), Szabó et al. [120] (surgeon trust), Moore et al. [226] (patient-centred metrics), Soomro et al. [228] (learning curves), Zolfagharian et al. [202] and Miller et al. [203] (runtime safety monitoring).

### 10.1. Multi-Stage Validation and Autonomy Levels

The progression toward Level 5 ‘Full Autonomy’ in surgery requires a systematic validation ladder. Yang et al.’s six-level framework [20] remains the field’s primary reference point for both research and regulation. Lee et al. [22] provide the most rigorous empirical mapping: reviewing 49 cleared surgical robots (2015–2023), they confirm 86% remain at Level 1 (Robot Assistance), only 6% reach Level 3 (Conditional Autonomy), and no Level 4–5 devices have been clinically deployed. Vasey et al. [121] conducted a comprehensive scoping review of intraoperative AI in robotic surgery (Annals of Surgery), finding that current applications are almost entirely preclinical mostly in silico or ex vivo, with only a few porcine in vivo experiments and no human RL trials.

Long et al. [13] represent the highest-autonomy validated surgical RL system to date, achieving generalised task autonomy in laparoscopic surgery across different platforms (Science Robotics, 2025). The SRT-H framework [138] achieved 100% success across eight ex vivo porcine specimens for the autonomous clipping and cutting phase of cholecystectomy, operating fully autonomously without human intervention, demonstrating readiness of hierarchical RL architectures for the next validation tier [229]. Validation follows a staged ladder consistent with the IDEAL Robotics framework [137] and the risk-stratification framework adopted by the IDEAL Robotics Colloquium.

Data are represented in the table below; prevalence estimates represent the authors’ qualitative synthesis of the surgical RL corpus, informed by general safe RL surveys [40,110]. They are indicative and should not be interpreted as meta-analytically derived statistics. ‘Rare’ = <3% of applied surgical RL studies; ‘Emerging’ = appearing 2022 onwards.

### 10.2. Runtime Monitoring and Safety Mechanism Prevalence

While the technical RL literature focuses on policy performance benchmarks, the integration of safety–critical runtime architectures remains strikingly low across the field. Based on the authors’ corpus analysis, formal safety guarantees, such as Control Barrier Functions (CBFs), Lyapunov-based approaches, or runtime shielding appear in fewer than 3% of applied surgical RL studies, despite their theoretical maturity [110,111]. Two new architectures for runtime safety assurance have emerged: Zolfagharian et al. [202] present SMARLA (Safety Monitoring for Deep Reinforcement Learning Agents) using statistical model checking of runtime state observations to detect impending constraint violations; Miller et al. [203] demonstrate Optimal Runtime Assurance via RL itself: an RL-trained safety guardian switches to a backup controller when the primary policy approaches unsafe regions.

### 10.3. Regulatory Comparison and Ethical Liability

The primary regulatory barrier to clinical deployment of RL-based surgical systems is their classification as Software as a Medical Device (SaMD). O’Sullivan et al. [198] provide the foundational legal and ethical framework, identifying accountability, culpability, and ‘doctor-in-the-loop’ as the core pillars of an ethical baseline. Abulibdeh et al. [227] present a critical analysis of the FDA’s AI healthcare product approval pipeline, concluding that current oversight provides an ‘illusion of safety’ for adaptive AI systems.

In the United States, the FDA’s Predetermined Change Control Plan (PCCP) framework, finalised in December 2024 as binding guidance under FDORA §515C, now provides a structured pathway for ‘adaptive’ AI-enabled devices [135]. Manufacturers must specify planned modifications upfront, detailing a Description of Modifications, a Modification Protocol, and an Impact Assessment. In the European regulatory landscape, the EU Medical Devices Regulation (MDR, EU 2017/745) requires notified body review and new conformity assessment for significant algorithmic changes [66]. The EU Artificial Intelligence Act (Regulation (EU) 2024/1689) classifies AI-enabled surgical robots as high-risk AI systems, imposing dual obligations under both the MDR and the AI Act [136,160]. The MDCG guidance document 2025-6 clarifies that pre-determined changes may not constitute a substantial modification under certain conditions [161]. The regulatory distinctions between conventional and RL/ML-driven surgical robots are summarised in Table 15.

Ethically, the move toward autonomy introduces an acute ‘Liability Gap.’ The concept of the ‘moral crumple zone’ where the nearest human operator absorbs legal and moral responsibility for failures in a complex automated system was originally articulated by Elish [163] and has been applied empirically to surgical robotics. The iRobotSurgeon Survey, gathering 10,955 individual scenario responses from 2191 respondents across 78 countries, found that for Level 2 systems, the robot manufacturer was the most commonly identified responsible party in 63.4% of Level 2 scenario responses, where a technical malfunction causes patient harm [162]. For Level 3 systems, where most decision-making was taken by the surgical robotic system, no clear consensus emerged on responsibility allocation; surgeon responsibility at 39.5% confirmed a contested attribution problem that worsens with increasing autonomy [162].

### 10.4. Clinical Outcome Metrics and Surgeon Trust

A critical disconnect exists between the proxy metrics that RL systems optimise and the clinical outcome measures that determine real-world value. Most surgical RL studies optimise for task-completion proxies path length, completion time, instrument smoothness, or force profile that do not reliably correlate with clinically meaningful endpoints such as Estimated Blood Loss (EBL), anastomotic leak rate, length of hospital stay, or post-operative recovery time. Younes et al. [118] provide a systematic review of Clinically Relevant Performance Metrics (CRPMs) in robotic surgery, finding that CRPMs are rarely correlated with patient outcomes. Soomro et al. [228] document via systematic review that learning curves in robot-assisted surgery are heterogeneous and inconsistently defined. Moore et al. [226] conduct a needs assessment for patient-centred education and outcome metrics in robotic surgery, finding large knowledge gaps in patient understanding of robotic surgical risks.

Fostering surgeon trust requires more than high task-success rates. Explainable AI (XAI) techniques, including saliency maps, attention visualisation, and counterfactual explanations, are increasingly recognised as necessary for surgical RL agents to make their policy intent legible to the operating surgeon [21]. O’B.A. et al. [230] investigate human–robot synergy in high-stakes medical procedures, confirming that cognitive load distribution and trust calibration between surgical team members are critical mediators of safety outcomes. Szabó et al. [120] examine surgeon attitudes toward robotic surgery, finding that perceived reliability, designer reputation, and prior experience are primary drivers of acceptance, and that over-trust is as dangerous as under-trust.

### 10.5. Regulatory Architecture Alignment: The CBF–FDA Correspondence

A practically important structural correspondence has received insufficient attention in the surgical RL literature. The shielded-RL architecture, in which a formally verifiable Control Barrier Function (CBF) safety layer constrains a stochastic RL policy during execution [103,115], maps naturally onto the FDA’s Predetermined Change Control Plan distinction between locked algorithmic components and adaptive ones [135]. The CBF layer, operating as a hard constraint on unsafe state transitions, qualifies as a locked safety–critical component under the PCCP framework, while the RL policy layer occupies the adaptive tier permitted to update within pre-specified performance and safety bounds [190]. Research groups designing systems for regulatory submission should exploit this alignment deliberately: formalising the boundary between the invariant safety envelope and the trainable action policy in system architecture documentation would substantially reduce the evidentiary burden at regulatory review [185]. The EU AI Act’s dual-conformity obligation under both the MDR and the AI Act for high-risk AI systems [136] further reinforces the importance of this architectural decision; a formally verified safety layer provides the technical backbone for the conformity assessment that CE marking will require for any Level 3+ autonomous surgical system.

## 11. Discussions and Future Research

The transition of reinforcement learning (RL) from simulation-bound research to clinically relevant surgical autonomy is a structural shift rather than a purely algorithmic one. Transformer-based segmentation now exceeds Dice 0.90 on endoscopic benchmarks [76,77], GPU-parallelised simulation compresses training from weeks to hours [71], and hierarchical RL (SurgicAI, SRT-H) with LLM-augmented decomposition enables long-horizon tasks unattainable by flat policies [113,138], while shared autonomy formulations enable progressive transfer [152]. Vision–language–action (VLA) architectures ground surgical intent in natural language [49,126,141], yet three persistent gaps remain. First, the sim-to-real gap for deformable tissue is unresolved; domain randomisation and hierarchical architectures are only partial mitigations. Second, safety guarantees remain heuristic: constrained formulations exist [103,115] but appear in <3% of applied RL studies, and no RL-derived system has FDA clearance for autonomous execution (86% of cleared robots remain at Level 1 [22]). Third, evaluation relies on proxy metrics rather than patient-level outcomes; ex vivo and in vivo laparoscopic validations exist [13,138] but in vivo human autonomous RL execution is absent. Hence, surgical RL is no longer constrained by algorithmic sophistication but by integration across physics, safety, and clinical validation.

Four structural imperatives follow. First, the field must standardise benchmarks and a universally adopted surgical RL suite with frozen physics, seed reporting, and force-weighted safety metrics—because without it, the evidence base is invisible to regulation. Second, the safety adoption gap must close: formal safety tools exist [12,47,104,190,199] but incentives reward performance over safety; venues should require safety verification for clinical claims. Third, multi-disciplinary integration is essential: closing the sim-to-real gap requires biomechanics and materials science, not just ML. Fourth, the field must reframe its goal from full autonomy (Level 5) to active shared autonomy, where control authority is continuously negotiated between agent and surgeon [132,152]. This review has limitations such as no prospective protocol registration, a screening and quality assessment restricted to dual-independent consensus, a corpus of 220 studies overwhelmingly simulation/ex vivo (no in vivo human autonomous RL execution), and unassessed publication bias (15% arXiv preprints, insufficient paired outcomes for formal assessment). These factors warrant epistemic caution in narrative synthesis interpretation. The emerging RL paradigms and their clinical translational roadmap are summarised in Table 16.

## 12. Conclusions

This systematic review employs PRISMA methodology to comprehensively map the integration of perception, control, safety, and regulation in surgical RL—a holistic synthesis absent in prior literature. The principal finding is that surgical RL remains structurally incomplete, yet its theoretical foundations are sound, and the remaining bottlenecks are sufficiently well characterised to support credible translational roadmaps. Safe reinforcement learning is paradoxically mature yet unadopted. Constrained Policy Optimization and Control Barrier Functions have demonstrated preclinical efficacy, yet formal safety verification appears in only a small minority of applied studies. This deficit reflects incentive misalignment rather than technical barriers—the field prioritises performance metrics over safety assurance. Foundation models and vision–language–action architectures mark a structural shift. RT-2 and Med-Gemini demonstrate that large language models can ground surgical intent and encode procedural reasoning into embodied actions. This convergence enables generalist agents capable of real-time adaptation to patient-specific tissue properties, a transition toward embodied surgical intelligence unprecedented in the field. The multimodal sensing gap is now recognised as a unified architectural challenge. Transformer vision models, hierarchical RL decomposition, and language-conditioned policies have converged to integrate semantic intent with motor control—a novel contribution of this synthesis. Regulatory readiness has been underestimated. While no RL system has achieved clinical autonomous clearance, 86% of FDA-approved surgical robots operate at Level 1 autonomy, revealing an incremental pathway. Active Shared Autonomy—wherein control authority is continuously negotiated between surgeon and robot—emerges as the architecturally correct target for clinical deployment. The sim-to-real gap persists as the concrete technical bottleneck. Evidence frontiers, including SRT-H’s 100% ex vivo cholecystectomy completion and in vivo laparoscopic validation, demonstrate conditional autonomy on bounded subtasks, yet generalisation remains unresolved. This is fundamentally a physics and biomechanics problem. The field stands at a well-defined inflexion point. Four imperatives define the path forward: standardised benchmarking across cohorts, embedding formal safety certificates in prototypes, evaluation aligned with patient outcomes, and transparent shared autonomy. The trajectory is credible. What remains is disciplined engineering across robotics, surgery, regulatory science, and patient advocacy. The work is clear, and the forward momentum is justified.

## Figures and Tables

**Table 1:** Principal Robotic Platforms for Surgical RL.

Platform	Deg. of Freedom	Control Interface	RL Compatibility	Primary Task Benchmarks	Autonomy Potential
dVRK	6–7	ROS-based API	Moderate	Suturing, Peg Transfer	Level 2 (Task)
Raven II	7	Open-source	High	Debridement, Navigation	Level 3 (Conditional)
KUKA LBR Med	7	Fast Robot Interface	High	Safe-RL, Collaborative Tasks	Level 3 (Safe-RL)
STAR	8+	Custom	High	Autonomous Anastomosis	Level 4 (High)

Note: Torque-level access enables Safe-RL applications where instrument-tissue forces are constrained to below 2 N [47].

**Table 2:** Major surgical datasets and benchmarks.

Dataset	Modality	Tasks/Labels	Size	SOTA Performance
Cholec80 [96]	Laparoscopy	Phase Recognition	80 videos/∼40 h	Accuracy: 90%+
EndoVis	Endoscopy	Tool/Anatomy Segmentation	Multi-challenge	Dice: 0.92–0.95
EgoSurgery-Phase [84]	Open Surgery	Phase/Gaze Recognition	15 h	Jaccard: +6.4%
SurgVU24 [97]	dVRK	Visual Understanding	840 h	Active Benchmark (2024)
JIGSAWS [57]	dVRK/ Laparoscopy	Gesture an Skill Assessment	∼9 h	Established baseline

**Table 3:** Hierarchy of surgical autonomy.

Level	Description	Example Task	Technical Notes
0	No autonomy	Manual control	Baseline teleoperation
1	Assistance	AI camera centring	Low-level autonomy; reactive
2	Task Autonomy	RL-based peg transfer/suturing	Sub-task policy; human-supervised
3	Conditional Autonomy	Robot executes; human intervenes for anomalies	Hierarchical RL (Manager–Worker)
4	High Autonomy	Multi-step procedure	Partial human oversight; real-time monitoring
5	Full Autonomy	Complete procedure	Not yet realised clinically

**Table 4:** Unified clinical metrics and evaluation framework for reinforcement learning in surgical robotics.

Category (with References)	Specific Metrics	Algorithm Target/ Evaluation Use
1. Technical Performance [53,112,113]	Task success rate, completion time, error count, partial-task score, composite weighted score	Baseline capability, hierarchical RL policies, task-specific benchmarking
2. Kinematic and Motion Quality [53,109,112,114]	Path length, motion smoothness (SPARC/LDLJ), instrument velocity/acceleration, endoscope smoothness	Motion planning, imitation learning, motor safety assessment
3. Safety and Constraint Adherence [47,109,115,116]	Force violations, anatomical boundary breaches, safety filter effectiveness, tool–tissue interaction force (mean/peak)	Safe-RL, constrained optimisation, safety shielding, haptic-enabled learning
4. Clinical and Surgical Competence [53,117,118]	GEARS, Global Rating Scale, Objective Performance Indicators, technical error classification, bimanual dexterity	Human-in-the-loop evaluation, clinical endpoint surrogates, proficiency benchmarking
5. Workflow and Phase Recognition [83,85,89]	Phase recognition F1-score, Jaccard index, step-transition accuracy, action recognition mAP	Temporal models (TCN, Transformer), hierarchical task decomposition
6. RL Algorithm and Learning Dynamics [109,115,116,119]	Sample efficiency, hyperparameter sensitivity, Sim-to-Real transfer rate, policy stability, constraint violation rate	Algorithm comparison (PPO, SAC, TD3, DDPG), domain randomization, Safe-RL
7. Human Factors and Cognitive Metrics [41,120,121]	Cognitive load (EEG, pupillometry, eye-tracking), workload (NASA-TLX), gaze patterns, muscle fatigue (EMG), trust/acceptance	Shared autonomy, Human–Robot Interaction, training assessment
8. Haptic and Force Safety [47,122,123,124]	Mean/peak interaction force, safe force range (2–5 N), stiffness mapping, force-feedback quality, image-to-force estimation	Haptic-enabled RL, sensorless force estimation, force-aware policy learning

**Table 5:** Reinforcement learning paradigms in surgical robotics.

RL Paradigm (with References)	Example Application	Safety Mechanism	Sim-to-Real Strategy	Compliance/ Regulatory Note
Constrained MDP/ Safe RL [47,110,115,145,149]	Tissue retraction, pedicle screw planning	CPO, CBF, Lyapunov, distance-based filters	N/A (often hardware-first)	ISO 14971, IEC 62304
Hierarchical RL [113,138,150]	Multi-step suturing	Sub-policy constraints	Progressive task decomposition	–
Sim-to-real (Domain randomisation) [11,46]	Blood suction, fluid handling	–	Domain randomisation, contrastive domain adaptation	Performance validation
Offline RL/LfD [99,146,147,148,151]	Policy initialisation from demonstrations	Reduces exploration risk on hardware	Sim-to-robot adaptation	Clinical efficacy standards
Human-in-the-loop/ Shared control [61,152]	Surgeon-assistive tasks, camera control	Virtual fixtures, confidence-based override	Real-time adaptation	User error prevention
Multi-agent RL [153,154,155]	Bimanual peg transfer, regrasping	Collision avoidance between arms	Transfer across dual arms	–
Model-based RL [14,156]	Soft robot control, tissue manipulation	Sample-efficient (reduces hardware fatigue)	Sim-to-real with augmented MDP	–
Scene-graph RL [142,143]	Tool–tissue relational reasoning	Encodes anatomical boundaries	N/A	–
Fluid-resistant RL [46]	Blood-robust suction	Safety-tested on dVRK	Domain randomisation	–

**Table 6:** Surgical RL algorithm family taxonomy.

Algorithm Family	Primary Algorithms	Contribution	Temporal Pattern	Typical Validation	Representative Evidence
Model-Free DRL [100,101]	PPO, SAC, DDPG+HER	Dominant (~60–70%)	Pre-2021 establishment; ongoing dominance in simulation benchmarks	Simulation only; limited lab	Richter 2019 [8]; Xu 2021 [55]; Scheikl/LapGym 2023 [72]; Schmidgall 2024 [71]
Demonstration-Guided/Offline RL [146,148,151]	CQL [99], BCQ, DEX, OTR	Growing (~10–15%)	Post-2022 surge; driven by data scarcity in surgical domain	Simulation + limited lab	Huang 2023 [148]: DEX across 10 SurRoL tasks; Zare 2023 [151]: OTR relabelling; Fazzari 2026 [175]
Safe/Constrained RL [12,103,104,115,190]	CPO, SER, CBF-RL, Recovery RL	Low-Moderate (~5–10%)	Post-2021 emergence; accelerating with regulatory pressure	Simulation; rarely benchtop	Fan 2024 [12]: SER reduced violations vs. baselines; Pore 2022 [47]: formal verification; Empleo 2025 [171]: near-zero CBF violations
Hierarchical RL (HRL) [113]	Manager–Worker, SurgicAI	Increasing (~10%)	Post-2022 adoption; decisive advantage over flat DRL for multi-step tasks	Simulation	Wu 2024 [113]: HRL >50% vs. flat DRL <10% suturing
Hierarchical IL (Language-conditioned) [138]	SRT-H (behaviour cloning + language planner)	Emerging	Achieves complex multi-step surgical tasks via IL decomposition distinct from RL	Ex vivo	Kim 2025 [138]: 100% ex vivo porcine cholecystectomy (SRT-H)
Model-Based RL [156,157]	DreamerV3, MBS, World Models	Sparse (<5%)	Emerging; one surgical-specific result (magnetic needle MBS)	Simulation + limited real	Barnoy 2022 [156]: 200× faster learning and successful real transfer for magnetic needle
VLA/Foundation Model RL [49,126,172,191]	RT-2, Med-Gemini, pi-0, LLM+DRL	Nascent (<3%)	Post-2023 emergence; no clinical deployment; highest potential, lowest maturity	Simulation/ concept-stage	Zargarzadeh 2025 [172]: LLM+DRL blood suction; Long 2025 [13]: multi-task learning; Schmidgall 2024 [17]: NMI framework

* Qualitative taxonomy based on the authors’ synthesis and cited survey-level classifications. The reported study counts provide descriptive context from the included corpus (N = 220) but do not constitute a formal quantitative prevalence analysis because studies may belong to multiple algorithm families. All performance findings are drawn from individual primary studies rather than pooled estimates.

**Table 7:** Surgical RL Studies—Task, Platform, Validation Level, Performance, and Transfer Strategy. Valid. Level: S = Simulation Only; LL = Limited Lab/phantom; DL = Diverse Lab/ex vivo; LR = Limited Real/in vivo animal.

Study	Task	Platform	Valid.	Key Metric	Reported Value	Transfer Strategy
Ou & Tavakoli 2023 [14]	Tissue point manipulation	dVRK (real)	LL	Success rate sim vs. real	∼100% sim → ∼73% real	FEM-based DR
Scheikl et al., 2022 [11]	Deformable tissue retraction	dVRK (real)	LL	Success rate (no retrain)	∼50%	Pixel-level GAN domain adapt.
Haiderbhai et al., 2025 [167]	Rope/suture cutting	dVRK (real)	LL–DL	Success rate (real)	90–97.5%	DR + reward shaping
Wu et al., 2025 (SurgicAI) [113]	Multi-step suturing	dVRK-style	LL	HRL vs. flat DRL	HRL > 50% vs. flat < 10%	Progressive decomposition
Kim et al., 2025 (SRT-H) [138]	Clipping an cutting	Ex vivo porcine	IL	Success across 8 specimens	100% (fully autonomous)	Language-conditioned hierarchical IL (not RL)
Fan et al., 2024 (SER) [12]	Tissue cutting an debridement	SurRoL sim	S	Constraint violations	Reduced vs. all baselines	Sim only
Empleo et al., 2025 [171]	Robotic suturing	Simulation	S	Violations under OOD	Near-zero	Sim only
Pore et al., 2022 [47]	Tissue retraction	dVRK sim	S	Prob. of unsafe config.	Substantially reduced	Formal verification
Barnoy et al., 2022 (MBS) [156]	Magnetic needle control	Physical robot	LL	Positional error: sim vs. MBS	3 mm → 1 mm	Model-based simulator
Lu et al., 2024 [46]	Autonomous blood suction	dVRK (real)	LL	Sim-to-real transfer	Successful transfer	DR (fluid/tissue params)
Mei et al., 2025 [168]	Endovascular guidewire nav.	Physical + sim	LL	Sim-to-real transfer	Transfer demonstrated	DR projections
Long et al., 2025 [13]	Multi-task laparoscopic	Multiple platforms	IL+RL+FM	Multi-task generalisation	Demonstrated in vivo	Cross-platform embodied intelligence
Zargarzadeh et al., 2025 [172]	Blood suction (LLM-DRL)	dVRK (sim)	S	Task planning + execution	Demonstrated	Sim only
Medany et al., 2024 [192]	Microvasculature navigation	Ultrasound microrobots	DL	Success Rate	90%	Model-Based RL (Dreamer v3)
Furnari et al., 2025 [193]	Multi-task (Suture, Knot, Needle)	dVRK (real)	LL	Success rate/Error	70%/0.5 cm	Sequence-based Imitation Learning

Note: This table is descriptive, not exhaustive. It includes studies reporting quantitative performance evidence suitable for structured comparison.

**Table 8:** Validation Maturity Distribution: Estimated corpus profile and representative performance data. (Tang et al. [16]).

Maturity Level (Tang et al. [16])	Est. Share *	Reported Performance (Individual, Not Pooled)	Primary Translational Bottleneck
Level 1: Simulation Only	~65–70%	85–100% for structured tasks (not directly translatable). LapGym reveals high sample complexity even for toy tasks [72].	Reality gap: rigid/PBD physics vs. visco-elastic tissue; no sensor noise, latency, or perfusion modelled
Level 2: Limited Lab (Phantom/Rigid Benchtop)	~15–18%	Ou and Tavakoli [14]: ~73% real vs. ~100% sim (~27 pp drop); rope cutting: 90–97.5% with DR [167]	Phantom material variability; sensor calibration across labs; geometric fidelity only, no biological variability [195]
Level 3: Diverse Lab (Ex vivo/Deformable Tissue)	~8–12%	Scheikl [11]: ~50% real-dVRK deformable retraction (domain adaptation, no retraining); SRT-H [138]: 100% ex vivo porcine (clipping + cutting, fully autonomous)	Altered post-mortem tissue mechanics; no perfusion or bleeding; biological decay artefacts; high cost [196]
Level 4: Limited Real (In vivo Animal)	~3–5%	STAR [197]: demonstrated in vivo soft-tissue suturing; Yao [48]: successful guidewire transfer; Long [13]: demonstrated in vivo multi-task	Full biological complexity; ethical approval; real-time monitoring; safety–critical hardware certification
Level 5: Diverse Real (Clinical/Human)	<2%	No RL autonomy data at this level. AquaBeam (Level 2 conditional autonomy, non-RL) is the only cleared autonomous tissue-removal device [22,23].	Regulatory clearance (FDA/CE); formal safety verification; IRB ethics; liability framework unresolved at Level 3+ [162,163,198]

* Qualitative approximations consistent with Tang et al. [16] and Vasey et al. [121]. Not meta-analytically derived. Individual performance figures from cited primary studies.

**Table 9:** Safety assurance mechanisms in surgical RL.

Mechanism	Evidence/Notes	Est. Adoption	Guarantee	Key Finding
Force/torque threshold [47,159,201]	Common safeguard; halts when forces exceed limits (2–5 N for grasping/retraction) [159,201].	~20–25% (most common)	No (heuristic)	Baseline comparator; insufficient for Level 3+ cert.
Kinematic anomaly detection [40,110,113]	Overrides when joint deviations exceed safety manifold; validated in hierarchical benchmarks [113].	~15–20%	No	Override on deviation; no formal bound.
Uncertainty/confidence estimation [110,171]	Bayesian/ensemble methods signal partial override when confidence is low [110].	~10–15%	No (probabilistic)	Ensemble + CBF; near-zero OOD violations.
Visual/OOD monitoring [110]	Image-based anomaly detection for OOD states in tool tracking/servoing.	~5–8%	Partial	OOD detection.
CMDP/CPO/SER [12,103,170]	SER reshapes unsafe actions into learned safe manifold; outperforms reward-penalty and CMDP baselines [12].	Low-moderate	Partial (SER bound)	SER >reward–penalty an CMDP.
Control Barrier Functions (CBF) [115,171,190]	Safety-certified control via barrier functions.	~3–5%	Yes (set invariance)	Near-zero dVRK violations; formal guarantee.
Lyapunov-based shielding [145,149,199]	Formal stability cert for safety-constrained optimisation.	~2–3%	Yes (stability)	Validated for pedicle screw planning.
Formal verification [47]	Probabilistic unsafe config estimation via Interval Analysis; most rigorous formal approach [47].	Rare (<3%)	Yes (probabilistic)	Most rigorous approach; tissue retraction domain.
Runtime safety monitors [202,203]	SMARLA (statistical model checking); RTA switches backup controller near unsafe regions [202,203].	Emerging (post-2022)	Bounded (statistical)	Model checking runtime states; DRL-applicable.
Human-in-the-loop [18,61,174]	Real-time GAIL-based supervision reduces catastrophic failures [61].	Low-moderate	No (human-governed)	GAIL correction accelerates policy learning.
Virtual fixtures [105]	RL + virtual fixtures projects unsafe actions into safe set [105].	Low-moderate	Partial (geometric)	RL + VF reduces unsafe interactions.

Estimated adoption values represent the authors’ qualitative, evidence-informed synthesis of the reviewed literature, guided by the survey-level classifications of Brunke et al. [111] and He et al. [110]. They indicate the relative maturity and adoption of safety mechanisms rather than quantitative prevalence based on a systematic study count. All paper-specific entries are drawn from the cited primary sources. ‘Rare’ denotes very limited adoption within the applied surgical RL literature, while ‘Emerging’ denotes relatively recent approaches in the literature.

**Table 10:** Paired sim-to-real performance evidence and HRL vs. flat DRL comparisons.

Source (Ref)	Task	Sim Perf.	Real/Ex Vivo	Gap (pp/mm)	Transfer Strategy	HRL vs. Flat
Ou & Tavakoli 2023 [14]	Internal tissue point manipulation	NR	∼1.3 mm	NR	FEM-based DR	Flat RL (SAC)
Scheikl et al., 2023 [11]	Deformable tissue retraction	100% success	50% success	50 pp	Pixel-level GAN adapt.	Flat RL
Haiderbhai et al., 2025 [167]	Rope/suture cutting	NR (high)	97.5% success (2D)	NR	DR + reward shaping	Flat RL
Kim et al., 2025 SRT-H [138]	Clipping an cutting (porcine)	NR	100% (8 specimens)	NR (ex vivo only)	Language-conditioned IL	HRL = 100% vs. Flat = 33.3%
Wu et al., 2025 SurgicAI [113]	Multi-step suturing	52% (simulation)	NR (simulation benchmark)	N/A	Hierarchical task decomp.	HRL (52%) vs. Flat (15%)
Yao et al., 2025 [48]	Autonomous guidewire navigation	90% success; mean error 3.53 mm (Target A)	100% success; mean error 2.92 mm (Target A)	+10 pp success; 0.61 mm absolute error difference	Domain Randomization + GAE	Flat RL (PPO)
Barnoy et al., 2022 MBS [156]	Magnetic needle control	31 mm error (pure sim)	6 mm error (MBS)	25 mm improvement	Model-based simulator	N/A
Long et al., 2025 [13]	Multi-task laparoscopic	NR	Demonstrated in vivo	NR	Visual parsing + visual servoing	Flat RL

A formal meta-analytic effect size estimate (Hedges’ g) for HRL vs. flat DRL requires identifying ≥3 papers reporting matched performance on identical tasks with compatible metrics. The current evidence base (Table 10) supports a consistent directional advantage for HRL in multi-step tasks but does not yet meet the threshold for pooled estimation.

**Table 11:** Algorithmic failure modes in surgical RL.

Failure Mode	Surgical Task	Clinical Observation	Severity (SRR SDE)	Detection Signal	Mitigation Strategy
Sparse Reward Collapse [119,148]	Suturing/ Knot-tying	Policy stagnates at episode start; zero progress after 10 k+ steps.	High (~80%)	Flat reward curve; near-zero success in early training.	Intrinsic Reward (IRPO [119]); HER; DEX demonstration-guided exploration [148].
Proxy Reward Hacking [32,103,151]	Needle passing/ Retraction	High-force ‘jerk’ movements satisfy distance proxy while damaging tissue.	Medium (~45%)	Unexpected force spikes; high tool velocity variance.	CPO [103]; Force penalties; OT-based reward relabeling (OTR [151]); RLHF [32].
Objective Drift [12,47,105,128]	Tissue retraction/Debridement	Tissue tearing; incremental reward leads agent off safe trajectory.	High (~70%)	Tissue deformation exceeds FEM safety threshold.	RLHF [128]; SER safe manifold projection [12]; Formal Verification [47]; Virtual fixtures [105].
Reward Over-optimisation [151,208]	Electrocautery/Long-horizon	Policy exploits loopholes (e.g., vibration) to accumulate reward.	Med–High (~55%)	Proxy reward high; true task completion near zero.	Reward capping; OTR [151]; Learning from suboptimal demos [208].
Exploration-Induced Harm [47,103,209]	Free-space navigation	Unconstrained stochastic exploration leads to irreversible contact with anatomy.	Critical (Irreversible)	Sudden force/torque spike beyond safety threshold.	CMDP [103]; Safety shielding [209]; Formal Verification [47].
Visual Domain Shift (Sim-to-Real)	Any vision-based task	Policy fails to recognise tools/anatomy due to glare, smoke, or blood.	High (~65%)	Low confidence in tool segmentation; visual “hallucinations.”	Surgical World Models; Domain Randomization; GAN-based translation.
Mechanical Hysteresis an Drift	Peg Transfer/Suturing	Commands result in “slack” or overshoot due to cable stretch in dVRK hardware.	Medium (~40%)	Increasing RMSE between joint encoders and visual tracking.	Hybrid Offline-Online RL; Digital Twin calibration; System Identification.

**Table 12:** Sim-to-real gap taxonomy for surgical RL.

Gap Type	Root Cause	Example Failure Scenario	TDM/Error Metric	Affected Platform	Proposed Mitigation
Visual/Rendering [67,95,102,206]	Photorealistic rendering mismatch; lighting/texture variation; surgical smoke [95]	Policy fails when specular highlights mask needle tip; smoke degrades segmentation	N/A (qualitative)	dVRK; endoscope-based platforms	NeRF-based digital twins; surgical desmoking [95]; Domain Randomization (DR); Multimodal fusion
Dynamics/Contact [51,52,156]	Non-linear cable friction/hysteresis; unmodelled contact physics	2–3 mm positional overshoot in peg transfer; tracking failure in pure simulation	∼2.5 mm mean error; 31 mm → 6 mm RMS reduction via MBS [156]	dVRK (cable-driven); magnetic robots	Model-Based Simulator (MBS) from real sensor data [156]; Sim-to-real cable stiffness calibration
Tissue Deformability [11,14,67,184]	Tissue morphology differs from rigid/particle sim; XPBD cannot model anisotropy [184]	Excessive retraction force causes unpredicted tissue tear; failure in point manipulation	>10 N contact force; ∼27 pp sim-to-real success gap [14]; 50% success in real [11]	All soft tissue interaction tasks	FEM-based DRL [14]; Position-Based Dynamics (PBD); GPU-accelerated FEM
Kinematics/Platform [73,74,210]	Legacy dVRK kinematic models differ from clinical robots; cable hysteresis	Policy trained on dVRK fails to generalise to da Vinci Xi kinematics	Qualitative; <5 transfer studies documented	dVRK → Xi transfer	Platform-agnostic RCM controllers; CRTK standardised API [74]; dVRK-Si hardware [73]
Task/Scenario Scope [2,67,72,174,211]	Narrow sim conditions lack clinical diversity; high sample complexity for toy tasks [72]	Overfitting to peg transfer with no transfer to multi-step clinical procedures	N/A (qualitative)	All platforms	Richer simulation (LapGym, ORBIT-Surgical); Human-in-loop data [61,174]; VR-enhanced data [211]
Sensing [52,67,212]	Stereo depth noise and calibration drift unmodelled in simulation	Depth estimation error cascades to incorrect tool–tissue proximity estimates	±1–3 mm stereo depth error [52]	dVRK ECM; stereo endoscopes	Sensor noise injection during training; Real2sim depth calibration; Tactile/Haptic augmentation [212]

**Table 13:** Surgical robotics platforms for RL research.

Platform	Open API	Research Access	Physics Engine	GPU	Key Capability	Primary Limitation
dVRK (1st gen) [8,55,210]	Yes (full)	High 40+ institutions	Rigid-body ROS/AMBF	No	Full sensor/actuator access; sim-to-real demonstrated	Hardware aging; cable-driven friction and hysteresis
dVRK-Si (2nd/3rd gen) [73]	Partial (custom firmware)	Emerging; released 2024	Rigid-body (extended)	No	Closer to clinical da Vinci S/Si kinematics; dVRK compat.	Bespoke electronics; proprietary embedded firmware
da Vinci Xi [74]	No	Very Low; clinical only	Proprietary	No	Gold standard in clinical practice (most procedures)	Fully proprietary; no low-level API; RL not permitted
Raven-II [74]	Yes (open source)	High 40+ institutions	Rigid-body (custom)	No	Open cable-driven design; programmable joint control	Cable-driven instability; limited sensor suite
SurRoL [55]	Yes (sim API)	High; open source	PyBullet	No	dVRK-compatible; 10 tasks; OpenAI Gym API	CPU-only physics; limited soft-body fidelity
LapGym [72,189]	Yes (sim API)	High; open source	FEM (SOFA)	No	Highest soft-body fidelity; 12 FLS-inspired tasks	Slow throughput; no native GPU parallelism
Surgical Gym [71]	Yes (sim API)	High; open source	Isaac Gym	Yes (native)	100–5000× speedup; high-throughput RL	No direct hardware equivalent; limited soft-body
ORBIT-Surgical [67,183]	Yes (sim API)	High; open source	PhysX (Isaac Sim)	Yes (8000+)	Photorealistic; 14 FLS tasks; dVRK + STAR support	Soft-body support limited; sim-to-real in progress
SurgicAI [113]	Yes (dVRK/ROS)	Moderate	PyBullet/ AMBF	No	Hierarchical suturing; HRL success > 50%	Narrow task scope (suturing focus)
STAR [197]	Partial	Low; specialised labs	Custom	No	Autonomous soft-tissue suturing *in vivo*	Task-specific hardware; not generalisable
Skill Simulators [156,173]	No	Very Low; clinical	Various	No	Historical RL baseline; clinical familiarity	No high-throughput API; timing jitter issues

**Table 14:** Comparative analysis of surgical RL benchmarks.

Benchmark	Base Platform	Core Feature	Open Source	Real-Robot?	Limitations
SurRoL [55,67]	PyBullet	10 tasks, dVRK-compatible RL baselines, OpenAI Gym API	Yes	Yes (dVRK)	Limited tissue deformability; CPU-only physics; no GPU acceleration
LapGym [72,98]	SOFA/ SofaGym [189]	12 environments, deformable tissue, threading tasks, PPO baselines	Yes	Limited	High fidelity but slow throughput; sample-inefficient for RL
ORBIT-Surgical [67]	NVIDIA Isaac Sim	14 GPU-parallel tasks, photorealistic rendering, dVRK+STAR models	Yes	Limited (demonstrated)	Limited soft-body realism vs. SOFA; no full deformable RL training pipeline
SurgicAI [113]	AMBF/ROS	Hierarchical suturing with compositional subtasks and metrics	Yes	Yes (dVRK)	Narrow task scope; HRL significantly outperforms flat DRL
Surgical Gym [71]	Isaac Gym (GPU)	High-throughput multi-task RL training (100–5000× speedup)	Yes	Limited	No real deformable-tissue equivalence yet
LASANA [134]	Video-based	Laparoscopic skill assessment from video	Yes	N/A	Evaluation-only framework; not a control environment
SURREAL [224]	MuJoCo	Scalable distributed RL benchmark suite	Yes	Limited	Not surgical-specific; general RL reproducibility baseline

**Table 15:** Regulatory comparison for RL-based surgical robots.

Aspect	Level	Current Robots (Level 1–2)	RL/ML-Driven Autonomy (Level 2–3+)
FDA pathway	Level 1–2 → 2–3+	Mostly Class II, 510(k) predicate-based; most Level 1 robots cleared with limited clinical testing [22]	Likely De Novo/SaMD pathways for adaptive RL; PCCP framework (finalised Dec 2024) provides structured pathway for adaptive AI [135]; FDA critiqued for weak AI oversight [226]
Clinical evidence base	Level 1–2 → 2–3+	Limited clinical data; 86% of FDA-cleared surgical robots at Level 1; few Level 3 have trials; no Level 4–5 devices cleared [22]	RL currently almost entirely preclinical (IDEAL stage 0, mostly in silico/ex vivo); only a few porcine in vivo experiments; no human RL trials [94,203]
Oversight/Monitoring	Level 1–2 → 2–3+	Static performance focus; PSUR required under EU MDR; limited post-market RL monitoring	Continuous monitoring required for adaptive algorithms; post-market drift surveillance; SMARLA-type runtime monitors for RL agents [228]; FDA AI/ML lifecycle guidance [135]
Liability/Accountability	Level 1–2 → 2–3+	Primarily surgeon + manufacturer; ‘doctor-in-the-loop’ legal baseline well-established [16]	Shared accountability; unresolved culpability as autonomy increases; iRobotSurgeon survey: manufacturer most responsible for Level 2 technical failures (63.4%); surgeon responsibility at 39.5% for Level 3; no consensus for Level 3 overall [162]; ‘moral crumple zone’ dynamic [163]
EU AI Act/MDR	Level 1–2 → 2–3+	EU MDR (2017/745) primary instrument; AI Act (2024/1689) dual obligations; MDCG 2025-6 clarifies interplay [161]	RL surgical robots = high-risk AI under AI Act; dual conformity assessment required [136,160]; data governance, transparency, human oversight mandated; liability frameworks under both instruments remain contested

**Table 16:** Emerging RL paradigms clinical translational roadmap.

Paradigm	Near-Term Tech Anchor	Main Clinical Gap	Timeline
Model-Based RL [55,71,156,157,174]	MBS for magnetic needle (acceleration >100× [156]); DreamerV3 world models [157]	Lack of benchmarks for complex soft tissue; visco-elastic fidelity	Short–Medium (1–3 yr)
Offline RL from Surgical Logs [99,146,151,175,231,232]	OTR relabelling [151]; ISAR offline manipulation [231]; CQL/BCQ [99,146]	Sparse/unlabeled clinical datasets; multi-site distributional mismatch	Short–Medium (2–4 yr)
Safe RL + Formal Verification [12,47,104,105,111,190,199,233]	Safe-DRL tissue retraction [47]; SER safe manifold [12]; Virtual Fixtures [105]	Verification confined to subtasks; no end-to-end clinical workflow guarantees	Medium (3–5 yr)
Meta-RL an Patient Personalization [233,234,235]	Federated DRL for policy selection [233]; Offline meta-RL for insertion [234]	No explicit meta-RL on patient morphology; privacy vs. adaptation trade-off	Medium–Long (4–6 yr)
Foundation Models/VLA Integration [11,36,49,126,174,191,236]	RT-2/Med-Gemini/$\pi_{0}$ VLA backbones [49,126,191]; Visual sim-to-real [11]	Regulatory auditability of VLAs; lack of “open-world” surgical controllers	Long (>5 yr)
Hierarchical RL [33,113,138]	SurgicAI HRL (>50% success [113]); SRT-H lang-conditioned HRL (100% ex vivo [138])	Inter-level credit assignment; coverage of multi-step procedures	Medium (3–5 yr)
Active Shared Autonomy [132,148,152,174,208,211]	Confidence-weighted blending [152]; DEX demonstration-guided exploration [148]	Principled real-time fusion of human intent and RL policy	Medium (3–5 yr)

## Data Availability

No new datasets were generated or analysed during this study. All evidence supporting this review is derived from the published primary studies cited herein, which are publicly available through their respective journals and repositories. The completed PRISMA 2020 checklist is provided as Supplementary Material S1. Database-specific search strategies are reported in Section 2.3. Extracted study-level data are presented in Table 6, Table 7, Table 8, Table 9 and Table 10 of the main manuscript.

## Supplementary Materials

The following supporting information can be downloaded at: https://www.mdpi.com/article/10.3390/biomimetics11080577/s1.

## Abbreviations

The following abbreviations are used in this manuscript:

**Abbreviation**	**Full Term**	**Abbreviation**	**Full Term**
AI	Artificial Intelligence	ACM	Association for Computing Machinery
AMSTAR	Assessing the Methodological Quality of Systematic Reviews	CBF	Control Barrier Function
CoA	Certificate of Acceptability	CPO	Constrained Policy Optimisation
CRTK	Collaborative Robotics Toolkit	CVaR	Conditional Value at Risk
DL	Deep Learning	DRL	Deep Reinforcement Learning
DR	Domain Randomisation	dVRK	da Vinci Research Kit [210]
EU AI Act	Regulation (EU) 2024/1689 on Artificial Intelligence	EU MDR	European Union Medical Device Regulation
FDA	US Food and Drug Administration	FLS	Fundamentals of Laparoscopic Surgery
GEARS	Global Evaluative Assessment of Robotic Skills	GRS	Global Rating Scale
HRL	Hierarchical Reinforcement Learning	HRI	Human–Robot Interaction
IEC	International Electrotechnical Commission	IEEE	Institute of Electrical and Electronics Engineers
IL	Imitation Learning	IQR	Interquartile Range
ISO	International Organisation for Standardisation	JIGSAWS	JHU-ISI Gesture and Skill Assessment Working Set
LfD	Learning from Demonstration	LLM	Large Language Model
MAML	Model-Agnostic Meta-Learning	MBS	Model-Based Simulation
MDCG	Medical Device Coordination Group	MDP	Markov Decision Process
MIS	Minimally Invasive Surgery	ML	Machine Learning
NASA-TLX	NASA Task Load Index	NLM	National Library of Medicine
OPI	Objective Performance Indicator	ORBIT	Isaac Orbit Simulation Framework
PBS	Position-Based Simulation	PPO	Proximal Policy Optimisation
pp	Percentage Points	PRISMA	Preferred Reporting Items for Systematic Reviews and Meta-Analyses
PROBAST	Prediction model Risk Of Bias Assessment Tool	RL	Reinforcement Learning
REML	Restricted Maximum Likelihood	SAC	Soft Actor–Critic
SER	Safe Experience Reshaping	sim-to-real	Simulation-to-real transfer
SOFA	Simulation Open Framework Architecture	SPARC	Spectral Arc Length
SRT-H	Surgical Robot Task Hierarchical Framework	STAR	Smart Tissue Autonomous Robot
SWIRL	Sequential Windowed Inverse Reinforcement Learning	TD3	Twin Delayed Deep Deterministic Policy Gradient
VLA	Vision–Language–Action	VLM	Vision–Language Model
XPBD	Extended Position-Based Dynamics		
