# Supplementary material for: Applications of Reinforcement Learning for Autonomous Surgical Robotics: A Systematic Review
Source: Biomimetics (Basel). 2026 Aug 12;11(8):577. doi: 10.3390/biomimetics11080577 (PMC13511724; doi:10.3390/biomimetics11080577)
Supplement: Supplementary file 1 [file biomimetics-11-00577-s001.zip › biomimetics-4406754-supplementary.pdf]

Supplementary S1

Applications of Reinforcement Learning for Autonomous Surgical Robotics: A Systematic Review

Table. S1 – PRISMA 2020 Checklist

**Section and Topic**	**Item No.**	**Checklist Item**	**Location in Manuscript**
TITLE	1	Identify the report as a systematic review	Title, p. 1
ABSTRACT	2	Structured summary including background, objectives, data sources, eligibility criteria, data extraction and synthesis methods, results, limitations, conclusions, and registration	Abstract, p. 1
INTRODUCTION – Rationale	3	Describe the rationale for the review in the context of existing knowledge	Section 1, pp. 1–4
INTRODUCTION – Objectives	4	Provide an explicit statement of the objectives or questions the review addresses	Section 1.1, p. 3; Section 2.1, pp. 4–5
METHODS – Eligibility criteria	5	Specify the inclusion and exclusion criteria and rationale	Section 2.4, p. 5
METHODS – Information sources	6	Specify all sources searched	Section 2.3, pp. 4–5
METHODS – Search strategy	7	Present the full search strategy	Section 2.3, pp. 4–5
METHODS – Selection process	8	Specify screening methods and reviewers	Section 2.5, pp. 5–6
METHODS – Data collection	9	Specify data collection methods	Section 2.6, p. 6
METHODS – Data items	10a	List and define all outcomes	Section 2.6, p. 6
METHODS – Data items	10b	List and define all other variables	Section 2.6, p. 6; Table 4

METHODS – Risk of bias	11	Specify risk-of-bias assessment methods	Section 2.7, pp. 6–7
METHODS – Effect measures	12	Specify effect measures	Section 2.8, p. 7; Section 7.4, pp. 22–24
METHODS – Synthesis methods	13a–13f	Eligibility, preparation, tabulation, synthesis, heterogeneity, and sensitivity analyses	Section 2.8; Tables 7–10; Sections 7.4–7.5
METHODS – Reporting bias	14	Methods used to assess reporting bias	Section 7.6, pp. 24–25
METHODS – Certainty assessment	15	Methods used to assess certainty of evidence	Section 2.7
RESULTS – Study selection	16a	Describe search and selection results	Section 2.5, pp. 5–6
RESULTS – Study selection	16b	Report exclusions and reasons	Figure 1; Section 2.5, p. 6
RESULTS – Study characteristics	17	Present characteristics of included studies	Tables 6–10
RESULTS – Risk of bias	18	Present risk-of-bias assessments	Section 2.7; Section 7.6
RESULTS – Individual studies	19	Summarise results for each study	Tables 7, 10
RESULTS – Syntheses	20a–20d	Present synthesis results and heterogeneity analyses	Section 7; Tables 8–9
RESULTS – Reporting biases	21	Assess risk due to missing results	Section 7.6, pp. 24–25
RESULTS – Certainty of evidence	22	Present certainty assessments	Section 2.7; Table 8
DISCUSSION	23	Interpret results in context	Section 11, pp. 37–39
DISCUSSION	24	Discuss limitations	Section 11, pp. 38–39
DISCUSSION	25	Conclusions and implications	Section 12, pp. 39–40
OTHER INFORMATION	26a	Registration information	Section 2.2
OTHER INFORMATION	26b	Protocol accessibility	Section 2.2
OTHER INFORMATION	27	Support and funding	Acknowledgements, p. 39
OTHER INFORMATION	28	Competing interests	Conflicts of Interest, p. 39
OTHER	29	Availability of data,	Data Availability

INFORMATION		code, and materials	Statement
